# Developing and Standardizing a Protocol for Quantitative Proton Nuclear Magnetic Resonance (^1^H NMR) Spectroscopy of Saliva

**DOI:** 10.1021/acs.jproteome.7b00847

**Published:** 2018-03-13

**Authors:** Alexander Gardner, Harold G. Parkes, Guy H. Carpenter, Po-Wah So

**Affiliations:** † Department of Mucosal and Salivary Biology, Dental Institute, King’s College London, London SE1 9RT, United Kingdom; ‡ Institute of Cancer Research, London SW7 3RP, United Kingdom; § Department of Neuroimaging, Institute of Psychiatry, Psychology and Neuroscience, King’s College London, Maurice Wohl Clinical Neuroscience Institute, 5, Cutcombe Road, London SE5 9RX, United Kingdom

**Keywords:** saliva, NMR, metabolomic profiling, protocol standardization

## Abstract

Metabolic profiling by ^1^H NMR spectroscopy is an underutilized technology in salivary research, although preliminary studies have identified promising results in multiple fields (diagnostics, nutrition, sports physiology). Translation of preliminary findings into validated, clinically approved knowledge is hindered by variability in protocol for the collection, storage, preparation, and analysis of saliva. This study aims to evaluate the effects of differing sample pretreatments on the ^1^H NMR metabolic profile of saliva. Protocol considerations are highly varied in the current literature base, including centrifugation, freeze−thaw cycles, and different NMR quantification methods. Our findings suggest that the ^1^H NMR metabolite profile of saliva is resilient to any change resulting from freezing, including freezing of saliva prior to centrifuging. However, centrifugation was necessary to remove an unidentified broad peak between 1.24 and 1.3 ppm, the intensity of which correlated strongly with saliva cellular content. This peak obscured the methyl peak from lactate and significantly affected quantification. Metabolite quantification was similar for saliva centrifuged between 750*g* to 15 000*g*. Quantification of salivary metabolites was similar whether quantified using internal phosphate-buffered sodium trimethylsilyl-[2,2,3,3-^2^H_4_]-propionate (TSP) or external TSP in a coaxial NMR tube placed inside the NMR tube containing the saliva sample. Our results suggest that the existing literature on salivary ^1^H NMR will not have been adversely affected by variations of the common protocol; however, use of TSP as an internal standard without a buffered medium appears to affect metabolite quantification, notably for acetate and methanol. We include protocol recommendations to facilitate future NMR-based studies of saliva.

## Introduction

Saliva is a useful fluid for biomedical analysis due to its inherently simple, noninvasive collection and its diverse composition, constituting both host and microbial DNA, RNA, proteins, peptides, and metabolites.^1^,^2^ In the past decade, considerable advances have been made using saliva as a source of diagnostic and prognostic biomarkers;^3^−^5^ however, the translation of preliminary research findings into validated clinical recommendations is still largely unrealized at present.^6^ This is particularly true regarding use of salivary metabolites as biomarkers, where considerable work is necessary to standardize protocols across analytical platforms.^7^ Although the majority of metabolic analyses of saliva are performed using mass-spectroscopy (MS)-based platforms, preliminary ^1^H-NMR-based studies have been demonstrated to reveal metabolic changes for multiple diseases. These include oral conditions such as dental caries,^8^ periodontal disease,^9^ and Sjögren’s syndrome^10^ as well as systemic conditions such as dementia.^11^ Other studies of salivary metabolite composition have investigated effects of factors such as smoking,^12^ exercise,^13^ dietary standardization,^14^,^15^ gender, body mass index^16^ and diurnal effects.^17^ The influence of salivary metabolite composition has been studied in the context of gustation^18^ and nutrition.^19^ Forensic applications of salivary ^1^H NMR metabolite profiling have also been investigated.^20^,^21^

**Figure 1:**
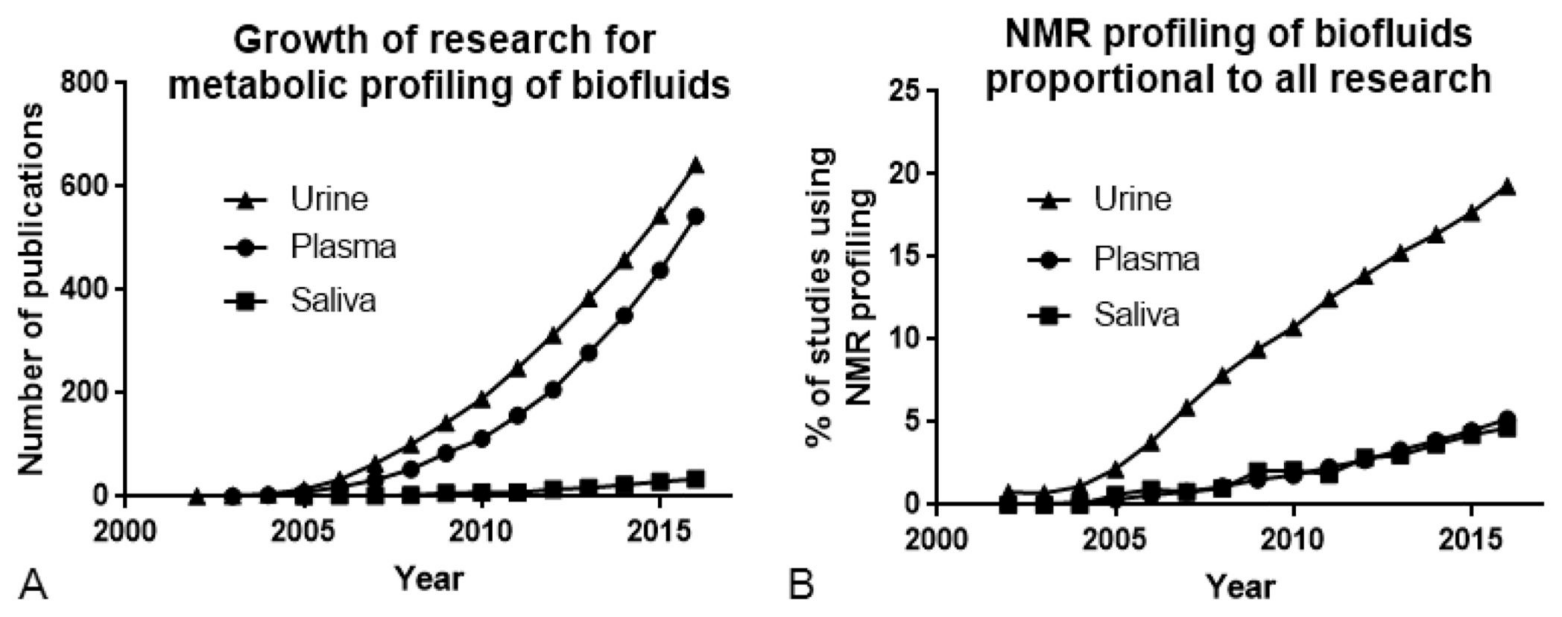
(A) Cumulative publications returned when searching “urine/plasma/saliva NMR metabolomics” in Web of Science by year. (B) Number of NMR-based metabolomic studies as a percentage of total research concerning the relevant biofluid (studies featuring “human urine/plasma/saliva” in the title).

Metabolic profiling of saliva by ^1^H NMR spectroscopy is underutilized compared with profiling of plasma and urine. This disparity is illustrated in Figure 1A, in which the number of publications returned by the Web of Science search engine for the terms “urine/plasma/saliva NMR metabolomics” by year and the proportion of NMR-based metabolomic studies as percentage of total publications in the field are shown. Figure 1B reveals that the proportion of NMR profiling studies of plasma and saliva is comparable; however, methodology in the former is considerably more established.

^1^H-NMR-based metabolic research of urine and plasma is facilitated by the availability of validated and published guidelines encompassing the collection, storage, preparation, and analysis of these biofluids.^22^,^23^ These guidelines achieve two significant goals: First, new researchers to the field can confidently undertake research by following these specifications, and, second, comparison of results between different studies can be readily made. Consequently, research findings for ^1^H NMR metabolite profiling of plasma and urine are rapidly approaching translation into clinical recommendations.^24^,^25^

No two studies of saliva by ^1^H NMR spectroscopy follow the same protocol. A selection of protocols is presented in Table 1, highlighting the degree of variability that exists in the current literature.

Protocol variability concerns several key aspects of sample preparation including centrifugation force and whether centrifugation was performed before or after initial freezing. The need to centrifuge saliva to remove cellular content (including host epithelial cells, leucocytes, and bacterial cells) before analysis is widely reported.^28^ Centrifugation has been shown to significantly affect the rheological and lubricant properties of saliva;^29^,^30^ however, there are no formal studies of centrifugation effects on the ^1^H NMR metabolic profile of saliva.

Freezing of saliva has been shown to alter the protein composition measured by MS and gel electrophoresis due to precipitation of salivary proteins.^28^,^31^ No literature exists on the effects of freeze−thaw events on salivary metabolite concentration measured by ^1^H NMR. A second unknown consideration regarding freeze−thaw processes is whether any difference occurs due to freezing whole-mouth saliva (WMS) before centrifugation (i.e., with cellular component present) or freezing supernatant following centrifugation. As shown in Table 1, both methods have been adopted.

Another protocol consideration with potential to greatly impact the data obtained from ^1^H NMR spectra of saliva is the method of quantification. To quantify metabolites in absolute terms, the use of an NMR standard of known concentration is required. The majority of studies on saliva use TSP (sodium trimethylsilyl-[2,2,3,3-^2^H_4_]-propionate) as a standard, directly mixed with the sample fluid. Such a practice is already known to be inappropriate for plasma as TSP binds to protein and the resulting signal is broadened/reduced, leading to higher metabolite concentrations.^22^ It has been observed that the relatively low protein concentration in saliva compared with plasma may avoid this problem; however, this has not been assessed statistically.^17^ Furthermore, the addition of buffered or nonbuffered standards is a variable that has not been evaluated.

The present study was therefore designed to evaluate the effects of centrifugation, freeze−thaw, and quantification methods on quantification of typical saliva metabolites: how different centrifugation forces and durations, freeze−thaw effects (including freezing of supernatant, freezing of WMS, and four freeze−thaw cycles), quantification method, external standard in a coaxial NMR tube, internal buffered TSP, and internal nonbuffered TSP affect quantification. The quantified metabolites are listed in Table 2. By addressing these common protocol variables found in the current literature base of salivary ^1^H NMR analysis, an evidence-based standardized protocol for collection, storage, preparation, and analysis of saliva samples by ^1^H NMR will be proposed. Additionally, this study will determine the extent to which data from published literature can be reasonably compared where protocol variability is present.

## Experimental Section

### Saliva Collection

All research was conducted following approval from King’s College London ethics committee (HR-15/15-2508). Unstimulated WMS was collected into sterilized universal tubes. Saliva samples initially collected from participants who had eaten within 1 h of sample collection and before, during, and 2 h post exercise (10 min of running upstairs) were observed to modulate the ^1^H NMR spectra of saliva (see Supplementary Figures S-1−S-3 and Table S-1). Thus for this study participants were instructed to have refrained from eating, drinking, and any oral activity (chewing gum, smoking, undertaking oral hygiene procedures) in the hour preceding collection time. Saliva was collected from a total of 12 healthy volunteers (5 males, 7 females), ages 23−44, but sample numbers varied for different aspects of the study (detailed below). Despite an interval of 1 h before sampling, resonances from xylitol (present in some chewing gum) were still apparent in the spectrum, although they were not observed to adversely affect metabolite quantification (Supplementary Figure S-1). Timing of collection was standardized as far as possible to between 11:00 and 12:00 a.m. All saliva was kept on ice from the moment of expectoration.

### Reagents

Trypan blue, sodium trimethylsilyl-[2,2,3,3-^2^H_4_]-propionate (TSP), deuterium oxide (D_2_O), 5 mm and 3 mm outer diameter (OD) Bruker SampleJet NMR tubes, and glacial acetic acid were purchased from Sigma-Aldrich (Poole, Dorset, UK).

### Preliminary Determination of Appropriate Centrifugation Forces

Saliva (5 mL) was collected from 11 individuals and gently mixed to ensure homogeneity. Aliquots of 20 and 2 *μ*L were taken for counting eukaryotic and prokaryotic cells, respectively (see below). Samples were then divided into 1 mL aliquots and centrifuged at 330*g*, 750*g*, 1500*g*, 3000*g*, and 15 000*g* for 10 min at 4 °C. Following centrifugation, eukaryotic and prokaryotic cells were counted in the individual supernatants.

### Cell Concentration

Saliva (20 *μ*L) was mixed with 0.4% Trypan blue (20 *μ*L) and placed in a hemocytometer counting chamber and viewed under a light microscope (500× magnification). Eukaryotic cells were counted and classified as either oral epithelial cells (~50−70 *μ*m diameter with a round prominent nucleus) or leucocytes (~10−30 *μ*m diameter with pleomorphic nuclei).

Bacterial cells were counted by heat-fixing 2 *μ*L of the sample to a glass slide, Gram staining, and viewing at 1250× magnification. The ratio of the area of one field of view to the whole sample area was calculated, and stained bacterial cells were counted.

### Sample Preparation for ^1^H NMR Spectroscopy

#### Centrifugation

Approximately 6 mL of saliva was collected from eight volunteers. Samples were gently agitated to ensure homogeneity and kept on ice in all stages of preparation. Five aliquots were taken and either not centrifuged or centrifuged at 750*g*, 1500*g*, 3000*g*, or 15 000 *g* for 10 min at 4 °C. Samples were stored at −80 °C prior to analysis.

Saliva (500 *μ*L) was added to 5 mm OD NMR tubes, and a sealed coaxial 3 mm OD NMR tube containing 300 *μ*L of 1 mM TSP in 50:50 D_2_O/milli-Q water was placed inside the 5 mm tube. To calculate the relative volumes of solution in the 5 and 3 mm tubes read by the NMR receiver coil, a precalibration step was performed with 4 mM acetate in the 5 mm OD tube.

For each sample, residual eukaryotic cell concentration was counted as described above.

#### Freeze−Thaw Treatments

For investigation into freeze−thaw effects, 4 mL of unstimulated WMS was collected from six participants on the day of analysis, so that WMS could be analyzed without freezing. Four aliquots were prepared as follows: (A) − WMS (1 mL) was centrifuged at 15 000 *g* for 10 min at 4 °C and kept on ice. (B) Same as for A but the supernatant was frozen at −80 °C and thawed on ice prior to analysis. (C) 1 mL of WMS was frozen at −80 °C and thawed on ice, then centrifuged at 15 000*g* for 10 min at 4 °C and kept on ice prior to analysis, performed within 3 h of collection. (D) Same as for B but the aliquot underwent four freeze−thaw cycles prior to analysis. Samples were frozen at −80 °C and thawed on ice in a cold room at 4 °C. The freezing step was for 0.5 h, and the thawing took ~45 min. Samples were prepared with TSP in the 3 mm OD NMR tubes (for external quantification method), as described below.

#### Quantification Method

Residual saliva from the 15 000*g* aliquot was then subdivided for quantification method comparisons with both buffered and unbuffered internal TSP. Unbuffered samples were prepared by adding 60 *μ*L of 0.5 mM TSP in D_2_O to 240 *μ*L supernatant, and buffered samples were prepared in the same way, except the TSP was in phosphate buffer (0.2 M Na_2_HPO_4_, 44 mM NaH_2_PO_4_, in D_2_O, pH 7.4). Mean ± SD sample pH after buffer addition was 7.44 ± 0.08.

### ^1^H NMR Spectroscopy

#### Acquisition

One-dimensional ^1^H NMR spectra were acquired on a Bruker Avance III spectrometer (Bruker Biospin, Karlsruhe, Germany) operating at a proton frequency 700.2 MHz. Samples were kept in a refrigerated chamber at 277 K prior to analysis and analyzed at 298 K following a 5 min period for temperature equilibration. Spectra were acquired with a Carr−Purcell−Meiboom−Gill (CPMG) spin−echo pulse sequence with water presaturation to filter out broad macromolecule resonances, a total echo time of 64 ms, relaxation delay of 4 s, acquisition time of 2.32 s, and 256 transients collected with 64k data points following four dummy scans, with a spectral width of 20 ppm (−5 to 15 ppm). Spectra were also acquired for each sample using a NOESY pulse sequence (see Supporting Information, Figure S-4), but all quantification was performed on the CPMG data.

#### Spectral Processing

Spectra were analyzed in TopSpin 3.5 (Bruker BioSpin). A 0.3 Hz exponential line broadening function was applied before Fourier transformation and automatic phase correction. Baselines were inspected and polynomial baseline correction applied. Metabolite assignments were made using Chenomx NMR suite 8.2 (Chenomx Inc.), human metabolite database (http://www.hmdb.ca) and literature values. Metabolite peaks were manually integrated and quantified relative to the TSP peak in each spectrum. The metabolites listed in Table 2 were measured. Metabolites were quantified based on the ratio of the integral of a known assignment relative to the integral of the standard TSP peak. This ratio was then adjusted to account for the ratio of metabolite and TSP protons giving rise to the signals and the difference in the volume measured by the probe-head for the TSP in the central (coaxial) 3 mm NMR tube and the sample in the 5 mm NMR tube. The latter explains the need for the precalibration step with two standards of known concentration.^32^ The configuration of the tubes and the calculation used is illustrated in Figure S-5. Where the internal standard was used, proton ratios of the metabolite peak to the TSP peak were calculated and then multiplied by the dilution factor of the sample caused by the addition of the standard TSP solution. As the TSP is not in contact with any protein that may be present in the sample, quantification by this method is unaffected by macromolecular binding to TSP.

#### Statistical Analysis

Data were inspected for normality and analyzed by repeated measures ANOVA with Greenhouse−Geisser correction of sphericity and a Bonferroni posthoc pairwise comparison test in SPSS. In some instances, interindividual variation in metabolite levels resulted in significant differences being detected by ANOVA but posthoc tests failed to determine where the differences lay. To account for this external intervariability in metabolite concentrations, in the experiment to determine the effects of centrifugation, metabolite concentrations were normalized to the levels measured in the uncentrifuged samples.

## Results

### Centrifugation Effects on Cell Types in Saliva

**Figure 2:**
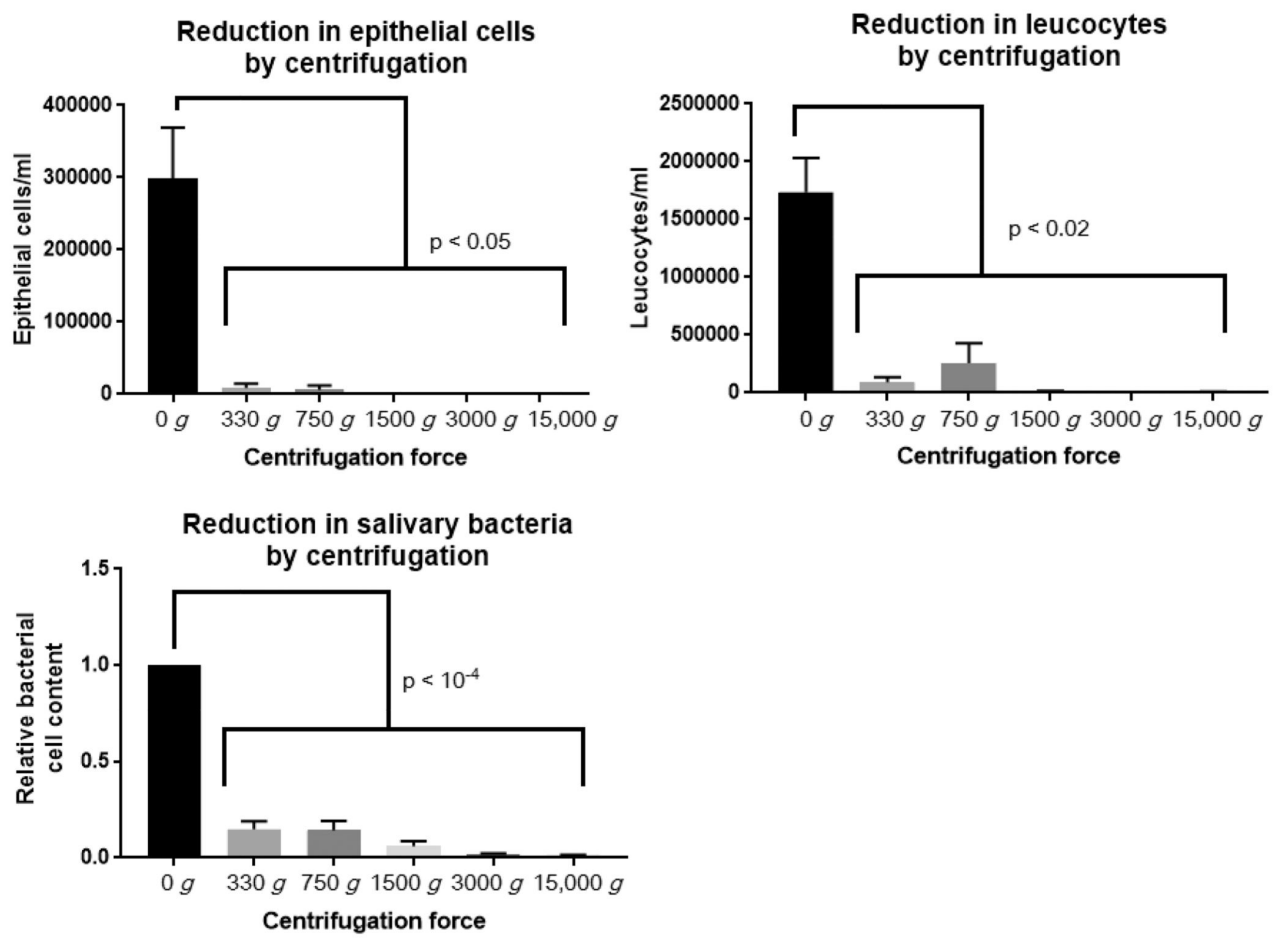
Reduction in epithelial cells, leucocytes, and bacterial cells in saliva following centrifugation (repeated measures ANOVA and Bonferroni posthoc test, *n* = 8). Bacterial cells are normalized to uncentrifuged levels due to large interindividual variation (between 2.91 × 10^7^ and 8.93 × 10^8^ cells/mL).

WMS contains abundant epithelial, leucocyte, and bacterial cells. Centrifugation significantly decreased the concentration of all cell types in saliva (Figure 2), but cell concentrations were similar irrespective of centrifugation speed applied. Thus centrifugation forces of 750*g*, 1500*g*, 3000*g*, and 15 000*g* were selected for investigation into effects on metabolite concentrations.

### ^1^H NMR Spectral Overview of Saliva

**Figure 3:**
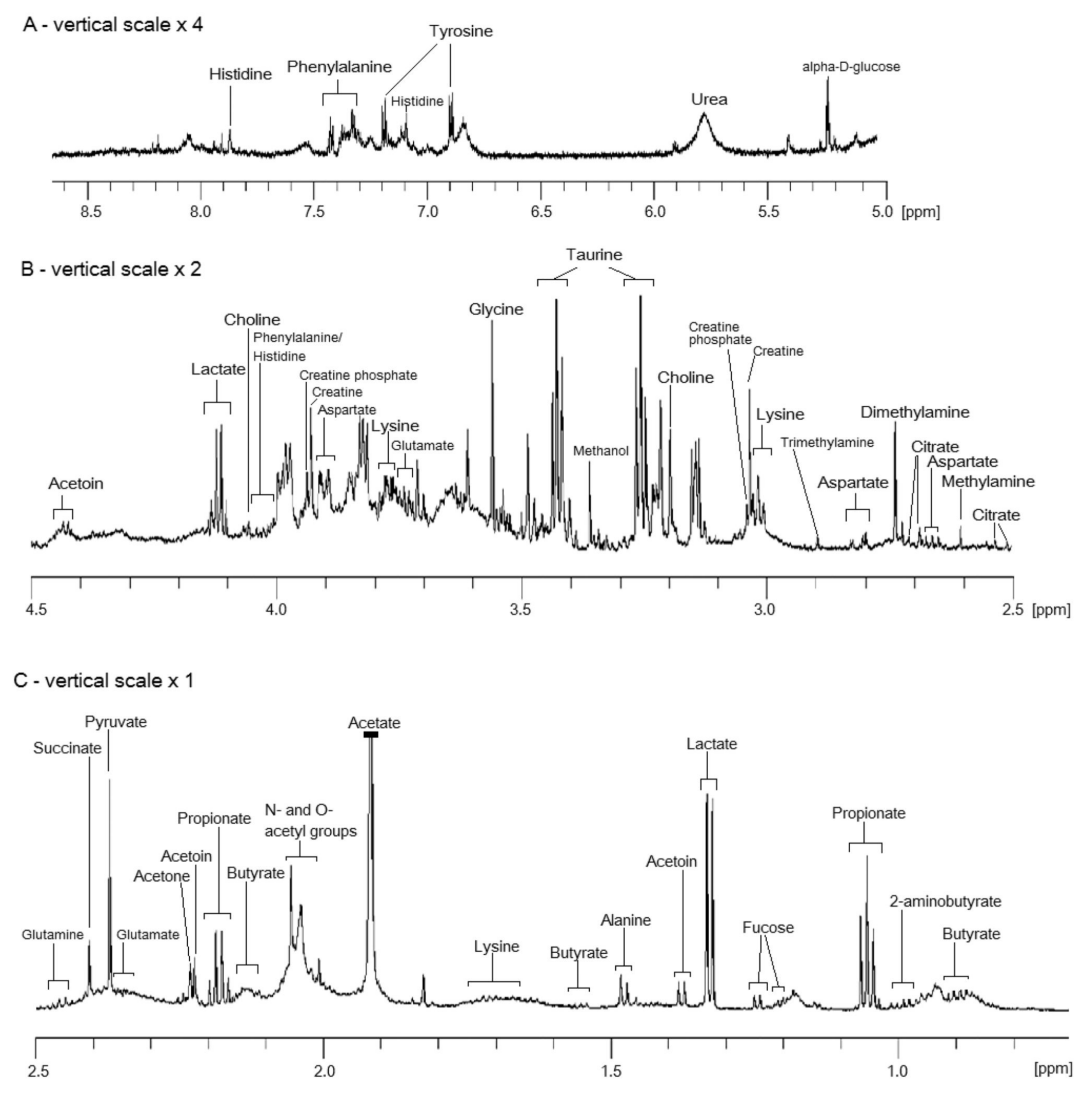
Representative 700 MHz 1D-CPMG ^1^H NMR spectrum (64 ms echo time) of saliva between 0.70 and 8.50 ppm. The residual water signal between 4.40 and 5.50 ppm has been removed. The vertical scale for the regions, 2.50−4.50 ppm and 5.00 to 8.50 ppm, has been doubled and increased by a factor of four times, respectively. Saliva was centrifuged at 15 000*g* prior to freezing, with quantification via external TSP in a coaxial tube. The acetate peak has been truncated.

A representative 1D ^1^H NMR spectrum of saliva is shown in Figure 3, and the assignments and concentrations of metabolites are summarized in Table 2. Metabolites typically observed in 1D ^1^H NMR spectra include organic acids (lactate, pyruvate, succinate, citrate), short-chain fatty acids (formate, acetate, propionate, butyrate), amino acids (tyrosine, histidine, phenylalanine, glycine, taurine), alcohols (methanol, ethanol), and amines (methylamine, dimethylamine, trimethylamine). The majority of the aforementioned metabolites are consistently reported in studies profiling salivary metabolites by ^1^H NMR. Additionally, we provide confirmation of the assignment of acetoin and refutation of the assignment of propylene glycol, both recently reported in saliva,^33^ via 2D ^1^H−^1^H COSY spectra (Figures S-6−S-8).

### Dietary and Physiological Modulation of ^1^H NMR Spectra of Saliva

Alterations in salivary metabolite composition were induced by both recent food consumption and exercise. These results are presented in detail in the Supporting Information. Notably, these included the presence of carbohydrate resonances obscuring other metabolite resonances when collecting saliva <1 h after eating (Figure S-1A), the effects of intraoral catabolism of dietary components (sucrose) on metabolites such as lactate (Figure S-2), and exercise causing a generalized increase in metabolite concentrations (Figures S-3A and S-3B).

### Sample Preparation Effects on ^1^H NMR Spectroscopy

#### Effects of Centrifugation Force

**Figure 4:**
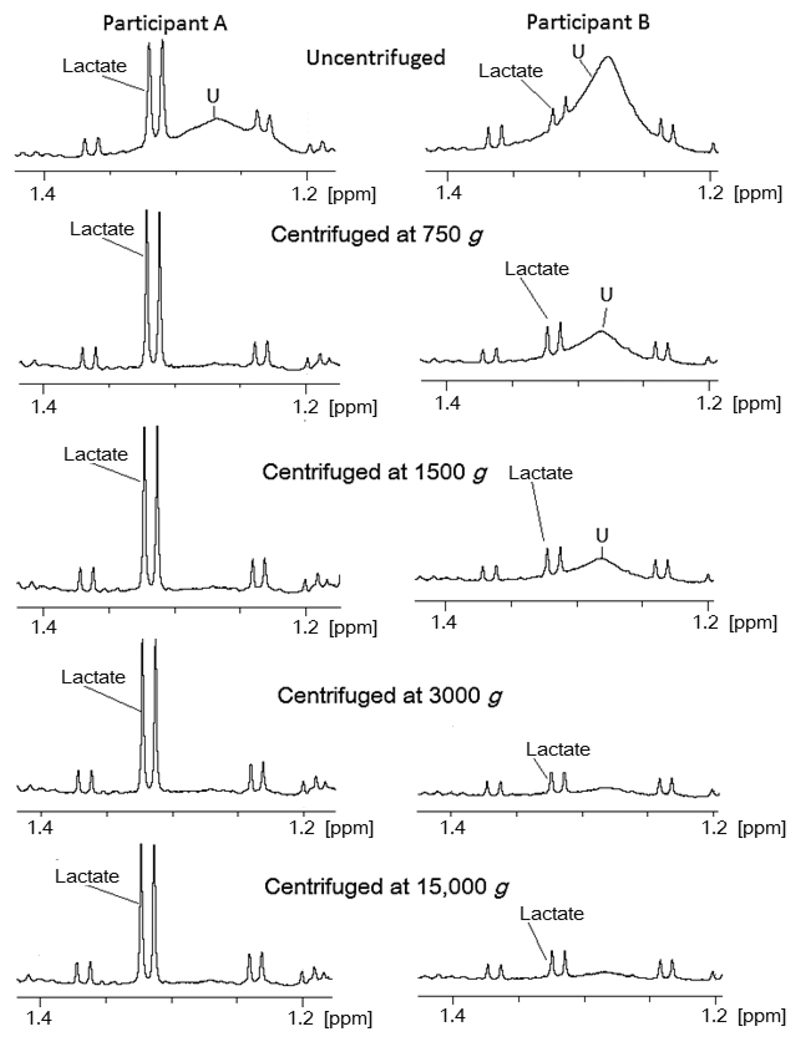
Partial 700 MHz CPMG ^1^H NMR spectra of samples from two participants (A,B). The unassigned broad peak (U) is removed by centrifugation at 750*g* for participant A; however, for participant B this peak persists with centrifugation at 750*g* and 1500*g*. Centrifugation at 3000*g* diminished peak U to the same extent as centrifuging at 15 000*g*. The superimposition of this peak on lactate is particularly noticeable in uncentrifuged samples. Samples were centrifuged at 15 000*g* prior to freezing, with quantification via external TSP in a coaxial tube.

**Figure 5:**
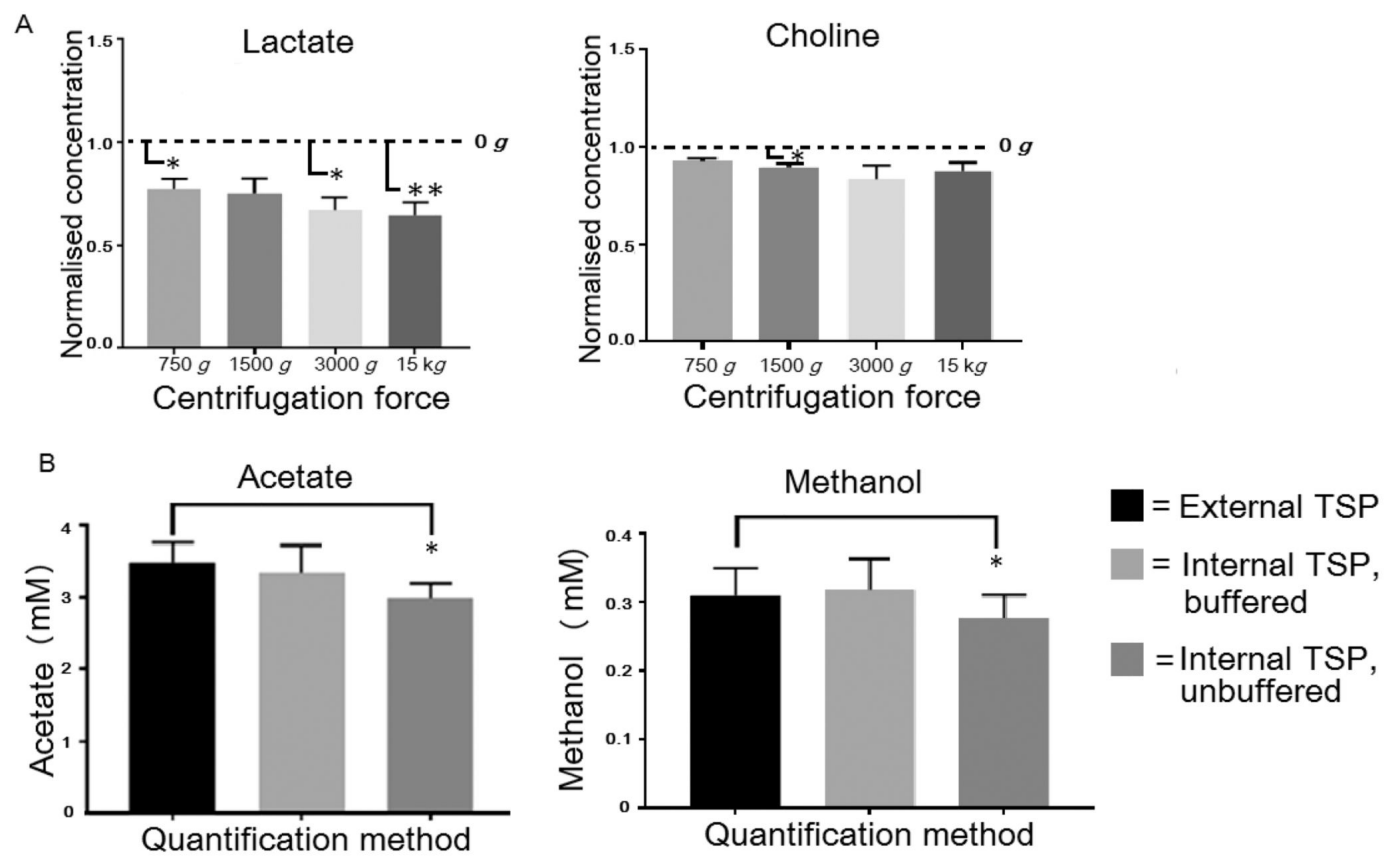
(A) Effects of centrifugation force resulting in significant differences in quantification of lactate and choline. Mean (±SEM) metabolite concentrations are shown normalized to uncentrifuged levels (horizontal dashed line, 0*g*) to account for interindividual variation, (*n* = 8). (B) Significant differences in quantification of acetate and methanol when measured by three different methods (*n* = 8). Significant differences detected by a Bonferroni posthoc pairwise comparison following repeated measures ANOVA with Greenhouse−Geisser correction of sphericity are denoted by * and ** (*p* < 0.05 and *p* < 0.01, respectively).

All spectra of uncentrifuged saliva consistently featured a broad resonance between ~1.24 and 3.0 ppm (labeled “U”, Figure 4), which overlapped the lactate doublet at ~1.32 ppm. In most cases, this peak was suppressed by the lowest centrifugation force (750*g*) but persisted in some cases, albeit diminishing with increasing centrifugation force. Lactate quantification was affected by the presence of U, with overestimation of the lactate concentration in the uncentrifuged aliquot compared with those subjected to centrifugation at 750*g*, 3000*g*, and 15 000*g* (*p* = 0.024, 0.012, and 0.008, respectively). The only other metabolites whose quantification changed as a result of centrifugation were choline and choline-containing compounds such as phosphatidycholine (PtdCho), with differences being detected only between tube. The acetate peak has been truncated. noncentrifuged samples and centrifugation at 1500*g* (*p* < 0.05; Figure 5A). ANOVA *p* values for acetoin and alanine were <0.05; however, posthoc testing failed to report a difference between the groups. In both cases, noncentrifuged samples had generally higher concentrations than centrifuged samples. Because of the proximity of these resonances to peak U, this likely reflects the same effects seen with lactate but to a lesser extent. Data are presented in Table S-2.

**Figure 6:**
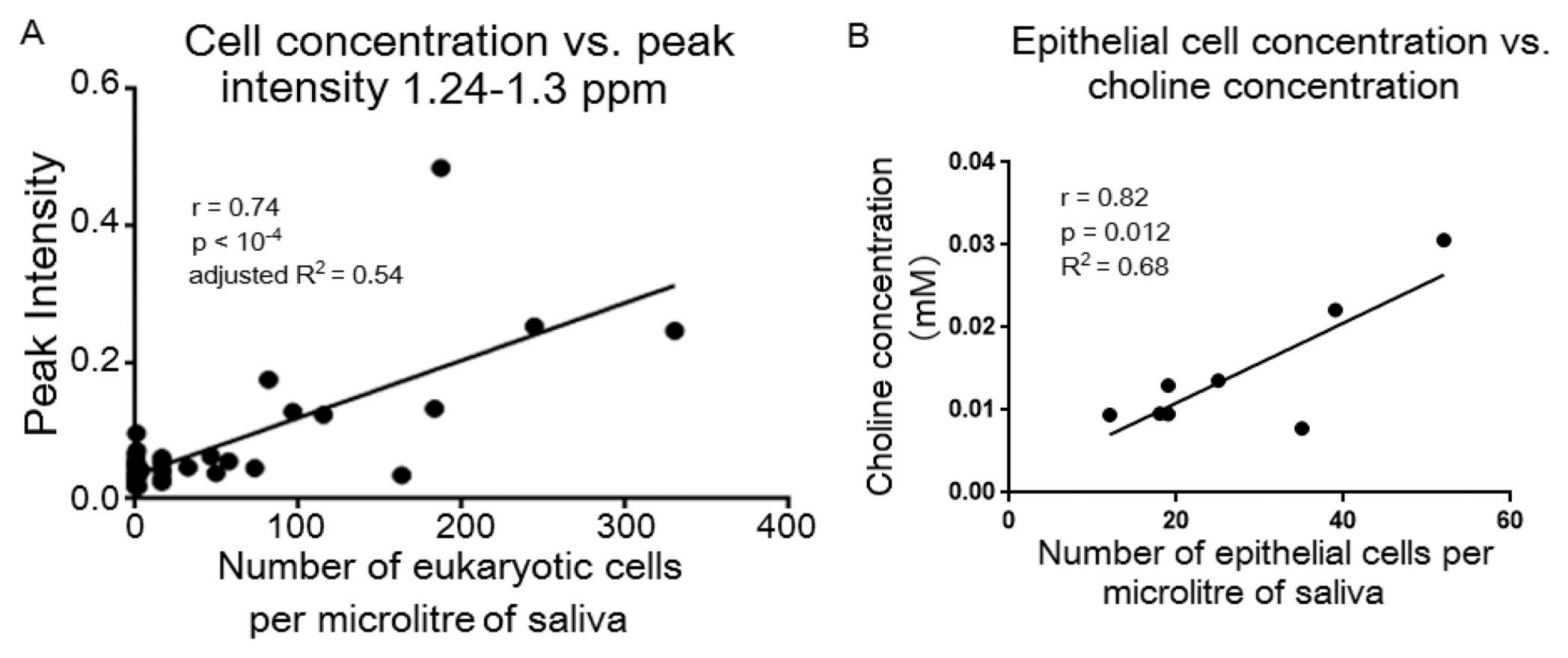
(A) Correlation between cell content and integral of region 1.24 and 1.30 ppm of the 700 MHz CPMG ^1^H NMR spectra of saliva uncentrifuged and centrifuged at 750*g*, 1500*g*, 3000*g*, or 15 000*g* before a single freeze thaw cycle and quantified using external TSP (*n* = 39). (B) Correlation between choline concentration and epithelial cell concentration in uncentrifuged saliva after a single freeze thaw cycle and quantified using external TSP (*n* = 8).

Subsequent analysis of the saliva showed a highly significant linear correlation (*p* < 10^−4^) between the total eukaryotic cell content and the integral of the region, 1.24 to 1.30 ppm, as a surrogate measure of U (Figure 6A). Peak U may therefore arise from lipidic aliphatic side chains of cell membrane components. Lipid side chains of lipoproteins contribute to a similar spectral peak in plasma spectra.^34^ Concentrations of choline and choline-containing metabolites were found to significantly correlate only with epithelial cells present in noncentrifuged samples (Figure 6B).

#### Effects of Freezing

Spectral profiles of centrifuged fresh saliva, saliva centrifuged prior to freeze−thawing, saliva centrifuged post freeze−thawing and centrifuged saliva subject to four freeze−thaw cycles were similar (Figure S-9). This was true for all samples. No significant differences were found between the different groups of sample treatments for any of the metabolites listed in Table 2. Data are presented in Table S-3.

#### Effects of Quantification Method

No significant differences in quantification were detected when quantifying via buffered internal TSP and external TSP in a coaxial tube. When quantification was performed using unbuffered TSP, significant differences were detected against external TSP for acetate and methanol (*p* < 0.05), Figure 5B. Data are presented in Table S-4.

## Discussion

The absence of methodological standardization has been identified as a primary cause of inconsistent results in the search for salivary proteomic and metabolomic biomarkers.^35^,^36^ This study proposes a standard protocol for 1D ^1^H NMR spectroscopy of saliva. Given that this field is relatively underexplored compared with genomic or MS proteomic profiling of saliva, early adoption of standardization is desirable. Additionally, with the variability in sample preparation for NMR analysis in the existing literature (see the Introduction), investigation into the effects of different sample preparation methods on salivary ^1^H NMR spectra can assist in comparing studies.

Despite the ubiquity of centrifuging saliva samples, there are relatively few studies examining the effects of centrifugation on subsequent ^1^H NMR analyses. For example, excessive centrifugation force has been recognized to cause membrane damage in many cell types, which could theoretically alter the ^1^H NMR metabolite profile of biological fluids.^37^,^38^ One study has investigated the effect of centrifugation on mass spectroscopy profiles of saliva, comparing forces of 1000*g* and 10 000*g*. The authors report that despite seeing differences in peak intensity, centrifugation had a “minimal effect”, although no formal statistical analysis of quantification was reported.^39^ The range of reported centrifugation forces for saliva is between 2000*g* and 15 000*g* (see the Introduction). Our findings show that quantifying acetate, lactate, or propionate in saliva centrifuged at 750*g*, 1500*g*, 3000*g* and 15 000*g* was comparable. Thus results from previous studies using such centrifugation forces are comparable. However, differences in quantifying lactate were observed if saliva samples were not spun due to the presence of an unassigned peak that was proportional to the cell content of samples. Thus centrifugation is necessary for the removal of cells to prevent this peak overlapping the lactate doublet at 1.32 ppm, leading to errors in quantification of lactate. This peak was seen to persist in one individual at forces up to 1500*g*. Given that high centrifugal force did not affect metabolite concentrations, whereas too low a centrifugation force may lead to residual cell contamination, centrifugation at 15 000*g* is advisable. Similarly, the higher content of choline and choline-containing compounds in noncentrifuged samples correlating with number of epithelial cells in the saliva may be of cellular origin given PtdCho is a membrane phospholipid.^40^

The ability to provide absolute and reproducible quantification of metabolites in a complex fluid environment with minimal sample preparation is a key strength of ^1^H NMR spectroscopy.^41^ Evaluation of NMR-based quantification of salivary metabolites is critical in validating the current literature base and influencing future studies. Use of TSP as an internal reference standard is the commonest approach to date. Such an approach has been cautioned for protein-rich biofluids such as plasma due to protein binding of the standard;^22^ however, for high-throughput spectroscopy, use of plasma buffer with internal TSP has been described.^23^ Silwood et al., in one of the earliest comprehensive ^1^H NMR analyses of saliva, describe minimal effects of protein binding due to the low protein content of saliva, although no quantitative comparisons were made. Our results find that, provided internal TSP is buffered with phosphate buffer (pH 7.4), quantification was comparable between internal TSP and external TSP in a coaxial NMR tube. This is of particular importance as using coaxial NMR tubes is less readily adaptable to a high-throughput approach or automated sample preparation.

Timing of freezing and the effects of repeated freeze−thaw cycles is another methodological variable for salivary ^1^H NMR spectroscopy that has not yet been assessed until this study. Freezing has been shown to alter the NMR spectra of plasma by broadening lipoprotein peaks compared with fresh samples.^42^ Furthermore, repeated freeze−thaw cycles have also been shown to alter plasma NMR spectra, particularly after the third cycle.^43^ Freezing of biofluids prior to analysis is almost always essential for logistical reasons. An important question to address with regards to saliva is whether samples can be frozen before centrifugation and removing the cell content. By analogy, freezing of whole blood prior to conversion into plasma or serum can result in hemolysis and leakage of intracellular metabolites from cells in the blood.^44^,^45^ Regarding saliva preparation, both approaches (freezing before and freezing after centrifugation) have been adopted for ^1^H NMR analysis (see the Introduction). Our results found no effects of freezing on either centrifuged or uncentrifuged saliva with respect to quantification of a number of metabolites compared with fresh (nonfrozen) supernatant. Additionally, repeated freeze−thaw cycles up to four times had no effect on quantifying metabolites. This knowledge is useful, for example, in studies where participants collect their own samples immediately upon waking;^15^ samples must be frozen before transport for processing in a laboratory setting.

An important consideration that all existing literature regarding saliva collection and preparation for ^1^H NMR analysis has in common is the need to keep samples chilled. Samples were kept at 4 °C or lower from the moment they were expectorated including during centrifugation, while thawing, and awaiting analysis in the NMR spectrometer. The use of metabolic inhibitors including sodium fluoride or sodium azide is described in the salivary ^1^H NMR literature.^15^,^17^,^46^ However, there is evidence that the introduction of sodium fluoride can alter the ^1^H NMR metabolite profile. MS analysis of oral biofilms has shown that sodium fluoride, an enolase inhibitor, results in an increase in 3-phosphoglycerate, albeit at levels below the detection threshold of ^1^H NMR.^47^ Sodium fluoride has also been shown to alter the citrate peaks of ^1^H NMR spectra of urine.^48^ Sodium azide, an inhibitor of cytochrome oxidase, has been shown to have no effect on the degradation of plasma lipoprotein at room temperature as lipolytic enzymes are not affected by azide.^41^ A study validating biobanking of urine and plasma for ^1^H NMR metabolomic studies recommends careful temperature control (<4 °C) of samples to inhibit cellular and enzymatic processes and cautions the addition of enzyme inhibitors to samples.^49^ Validation of saliva biobanking has revealed that maintaining saliva at 4 °C for 24 h before freezing causes minimal effect when compared with samples frozen immediately, although nitrite levels were found to decrease.^50^

A final protocol consideration for saliva collection prior to ^1^H NMR analysis concerns timing of collection with respect to both time of day and timing of other activities. Salivary flow and composition is under circadian control,^51^ and more recent evidence suggests that a minority of salivary metabolites displays circadian fluctuations.^52^ Collection should be standardized between participants as far as possible. The finding that the ^1^H NMR metabolite profile of saliva collected immediately on waking is significantly different from samples collected later in the day must also be taken into account.^15^ A range of exogenous substances have been reported in the ^1^H NMR spectra of saliva including dietary derived substances (e.g., aspartame, acesulfame-K, and caffeine) and substances from oral care products (e.g., chlorhexidine, xylitol, triclosan, and thymol).^17^ Most authors acknowledge the effects exogenous substances can have on the salivary ^1^H NMR spectrum by asking participants to abstain from ingesting substances prior to collection. We collected saliva at least 1 h after eating or having undergone other oral activities based on previous observations of carbohydrate levels in saliva. While this time period is somewhat arbitrary, 1 h proved to be sufficient for elimination of carbohydrate peaks obscuring salivary metabolites.

## Conclusions

Despite considerable variability in literature regarding preparation of saliva for ^1^H NMR metabolic profiling, our findings indicate that results are not likely to have been significantly altered by centrifugation parameters or freeze−thaw considerations. We demonstrate that previous study protocols quantifying metabolites in saliva by NMR spectroscopy using unbuffered internal TSP referencing are generally satisfactory for many metabolites, with the exception of acetate and methanol. We present an evidence-based protocol for preparation of saliva for ^1^H NMR metabolic profiling.

## Supplementary Material

The Supporting Information is available free of charge on the ACS Publications website at DOI: 10.1021/acs.jproteome.7b00847.

Supporting information

## Figures and Tables

**Table 1:** Summary of Protocol Variability in Existing ^1^H-NMR Spectroscopy Studies of Saliva^a^

	protocol consideration		
authors	sample storage	centrifugation	quantification method
Mikkonen et al., 2013^10^	transferred to lab on ice, centrifuged (a), stored −20 °C, defrosted, centrifuged (b)	(a) 3000*g* for 20 min at 4 °C (b) 10 000*g* for 5 min at 4 °C	internal phosphate-buffered TSP
Silwood et al., 2002^17^	transferred to lab on ice, centrifuged, stored −70 °C	unspecified	internal unbuffered TSP; external TSP in coaxial NMR tube
Wallner-Liebmann et al., 2016^15^	samples frozen at −20 °C, transferred to liquid nitrogen within 60 h; thawed at room temperature, centrifuged	14 000 rpm for 30 min at 4 °C	internal phosphate-buffered TSP
Takeda et al., 2009^12^	samples frozen at −80 °C, thawed, centrifuged	3000 rpm	internal unbuffered DSS
Dame et al., 2015^26^	samples centrifuged, stored at −20 °C then ultrafiltered (3 kDa filter)	10 000 rpm for 10 min	internal DSS in deproteinated samples
Neyraud et al., 2013^27^	samples centrifuged (a), stored at −80 °C, thawed and centrifuged (b)	(a) 15 000*g* for 30 min (b) 5000*g* for 10 min	internal TSP
Bertram et al., 2009^16^	transferred to lab at 4 °C, centrifuged, stored at −20 °C	2000*g* for 10 min	internal TSP

a TSP, sodium trimethylsilyl-[2,2,3,3-^2^H_4_]-propionate; DSS, 4,4-dimethyl-4-silapentane-1-sulfonic acid.

**Table 2:** Summary of Metabolite Assignments and Concentration Ranges in 700 MHz CPMG ^1^H-NMR Spectra of Saliva^a^

metabolite (HMDB number)	chemical shift (ppm) and multiplicity of characteristic resonances^b^	assignment	mean ± SEM salivary concentration (*μ*M)	range of salivary metabolite concentrations (*μ*M)
acetate (0000042)	1.92, singlet	CH_3_	3670.0 ± 236.0	2672.0–4780.0
acetoin (0003243)	1.37, doublet	CH_3_	43.1 ± 3.8	25.2–64.5
	2.21, singlet	CH_3_		
	4.42, quartet	CH		
alanine (0000161)	1.47, doublet	CH_3_	97.1 ± 13.5	49.2–207.4
	*3.76, quartet*	CH		
butyrate (0000039)	0.88, triplet	CH_3_	144.6 ± 11.8	87.0–196.9
	1.55, multiplet	CH_2_		
	2.14, triplet	CH_2_		
choline and choline-containing compounds (0000097)	3.18, singlet	CH_3_	21.1 ± 2.4	6.2–32.2
	3.51, *multiplet*^c^	CH_2_		
	4.07, *multiplet*^c^	CH_2_		
citrate (0000094)	2.51, doublet^d^	CH_2_	49.0 ± 6.4	24.9–103.9
	2.67, doublet^d^	CH_2_		
dimethylamine (0000087)	2.70, singlet	CH_3_	11.5 ± 1.7	4.6–23.9
ethanol (0000108)	1.17, triplet	CH_3_	98.2 ± 19.2	24.5–285
	*3.65, quartet*	CH_2_		
formate (0000142)	8.45, singlet	CH	102.6 ± 40.7	0.0–486.6
glycine (0000123)	3.54, singlet	CH_2_	172.6 ± 18.0	58.2–255.1
histidine (0000177)	*3.16, multiplet*	CH_2_	27.4 ± 5.3	5.6–61
	*3.23, multiplet*	CH_2_		
	*3.98, multiplet*	CH		
	7.09, singlet	CH		
	7.80, singlet	CH		
lactate (0000190)	1.33, doublet	CH_3_	224.9 ± 55.2	50.1–647
	4.1, quartet	CH_2_		
methanol (0001875)	3.34, singlet	CH_3_	32.9 ± 3.3	16.0–48.3
methylamine (0000164)	2.60, singlet	CH_3_	11.9 ± 0.8	6.9–15.9
phenylalanine (0000159)	*3.19, multiplet*	CH_2_	42 ± 5.7	20.1–80.4
	*3.98, multiplet*	CH		
	7.32, doublet	H2, 2′		
	7.36, multiplet	H3, 3′		
	7.42, multiplet	H4		
propionate (0000237)	1.05, triplet	CH_3_	517.4 ± 79.0	156.9–1039
	2.17, quartet	CH_2_		
pyruvate (0000243)	2.36, singlet	CH_3_	160.0 ± 19.3	56.8–262.3
succinate (0000254)	2.40, singlet	CH_2_	81.6 ± 16.4	26.7–205.5
taurine (0000251)	3.25, triplet	CH_2_	183.6 ± 29.2	90.8–396.2
	3.42, triplet	CH_2_		
tyrosine (0000158)	*3.02, multiplet*	CH_2_	42.0 ± 6.2	11.1–81.4
	*3.17, multiplet*	CH_2_		
	*3.92, multiplet*	CH		
	6.88, multiplet	H2, 2′		
	7.17, multiplet	H3, 3′		
trimethylamine	2.89, singlet	CH_3_	3.3 ± 0.5	1.3–7.2

a Metabolites are quantified in unstimulated saliva (after centrifugation at 15 000*g* and a single freeze-thaw cycle) with quantification using external TSP in a coaxial tube (*n* = 12). The Table does not include metabolites that can be qualitatively detected but are not reliably quantified due to superposition of other resonance frequencies.

b Resonances in italics are obscured in 1D ^1^H NMR spectra of saliva.

c These refer to chemical shifts for choline only.

d pH-sensitive chemical shifts.

## References

[1] Soares Nunes LA, Mussavira S, Sukumaran Bindhu O (2015). Clinical and diagnostic utility of saliva as a non-invasive diagnostic fluid: a systematic review. Biochem Med.

[2] Cuevas-Córdoba B, Santiago-García J (2014). Saliva: a fluid of study for OMICS. OMICS.

[3] Wong DT (2006). Salivary diagnostics powered by nanotechnologies, proteomics and genomics. J Am Dent Assoc, JADA.

[4] Lee Y-H, Wong DT (2009). Saliva: an emerging biofluid for early detection of diseases. Am J Dent.

[5] Zhang Y, Sun J, Lin C-C, Abemayor E, Wang MB, Wong DTW (2016). The emerging landscape of salivary diagnostics. Periodontol 2000.

[6] Yoshizawa JM, Schafer CA, Schafer JJ, Farrell JJ, Paster BJ, Wong DT (2013). Salivary biomarkers: toward future clinical and diagnostic utilities. Clin Microbiol Rev.

[7] Mikkonen JJW, Singh SP, Herrala M, Lappalainen R, Myllymaa S, Kullaa AM (2016). Salivary metabolomics in the diagnosis of oral cancer and periodontal diseases. J Periodontal Res.

[8] Fidalgo TK, Freitas-Fernandes LB, Angeli R, Muniz AM, Gonsalves E, Santos R, Nadal J, Almeida FC, Valente AP, Souza IP (2013). Salivary metabolite signatures of children with and without dental caries lesions. Metabolomics.

[9] Aimetti M, Cacciatore S, Graziano A, Tenori L (2012). Metabonomic analysis of saliva reveals generalized chronic periodontitis signature. Metabolomics.

[10] Mikkonen JJ, Herrala M, Soininen P, Lappalainen R, Tjäderhane L, Seitsalo H, Niemelä R, Salo T, Kullaa AM, Myllymaa S (2013). Metabolic Profiling of Saliva in Patients with Primary Sjögren’s syndrome. Metabolomics.

[11] Figueira J, Jonsson P, Nordin Adolfsson A, Adolfsson R, Nyberg L, Öhman A (2016). NMR analysis of the human saliva metabolome distinguishes dementia patients from matched controls. Mol BioSyst.

[12] Takeda I, Stretch C, Barnaby P, Bhatnager K, Rankin K, Fu H, Weljie A, Jha N, Slupsky C (2009). Understanding the human salivary metabolome. NMR Biomed.

[13] Santone C, Dinallo V, Paci M, D’Ottavio S, Barbato G, Bernardini S (2014). Saliva metabolomics by NMR for the evaluation of sport performance. J Pharm Biomed Anal.

[14] Walsh MC, Brennan L, Malthouse JPG, Roche HM, Gibney MJ (2006). Effect of acute dietary standardization on the urinary, plasma, and salivary metabolomic profiles of healthy humans. Am J Clin Nutr.

[15] Wallner-Liebmann S, Tenori L, Mazzoleni A, Dieber-Rotheneder M, Konrad M, Hofmann P, Luchinat C, Turano P, Zatloukal K (2016). Individual Human Metabolic Phenotype Analyzed by ^1^H NMR of Saliva Samples. J Proteome Res.

[16] Bertram HC, Eggers N, Eller N (2009). Potential of human saliva for nuclear magnetic resonance-based metabolomics and for health-related biomarker identification. Anal Chem.

[17] Silwood CJ, Lynch E, Claxson AW, Grootveld MC (2002). ^1^H and ^13^C NMR spectroscopic analysis of human saliva. J Dent Res.

[18] Mounayar R, Morzel M, Brignot H, Tremblay-Franco M, Canlet C, Lucchi G, Ducoroy P, Feron G, Neyraud E (2014). Salivary markers of taste sensitivity to oleic acid: a combined proteomics and metabolomics approach. Metabolomics.

[19] Morzel M, Neyraud E, Brignot H, Ducoroy P, Jeannin A, Lucchi G, Truntzer C, Canlet C, Tremblay-Franco M, Hirtz C (2015). Multi-omics profiling reveals that eating difficulties developed consecutively to artificial nutrition in the neonatal period are associated to specific saliva composition. J Proteomics.

[20] Harada H, Shimizu H, Maeiwa M (1987). ^1^H-NMR of Human Saliva. An application of NMR spectroscopy in forensic science. Forensic Sci Int.

[21] Grootveld M, Algeo D, Silwood CJ, Blackburn JC, Clark AD (2006). Determination of the illicit drug gamma-hydroxybutyrate (GHB) in human saliva and beverages by ^1^H NMR analysis. BioFactors.

[22] Beckonert O, Keun HC, Ebbels TM, Bundy J, Holmes E, Lindon JC, Nicholson JK (2007). Metabolic profiling, metabolomic and metabonomic procedures for NMR spectroscopy of urine, plasma, serum and tissue extracts. Nat Protoc.

[23] Dona AC, Jiménez B, Schäfer H, Humpfer E, Spraul M, Lewis MR, Pearce JT, Holmes E, Lindon JC, Nicholson JK (2014). Precision high-throughput proton NMR spectroscopy of human urine, serum, and plasma for large-scale metabolic phenotyping. Anal Chem.

[24] Duarte IF, Diaz SO, Gil AM (2014). NMR metabolomics of human blood and urine in disease research. J Pharm Biomed Anal.

[25] Mora S (2009). Advanced lipoprotein testing and subfractionation are not (yet) ready for routine clinical use. Circulation.

[26] Dame ZT, Aziat F, Mandal R, Krishnamurthy R, Bouatra S, Borzouie S, Guo AC, Sajed T, Deng L, Lin H (2015). The human saliva metabolome. Metabolomics.

[27] Neyraud E, Tremblay-Franco M, Gregoire S, Berdeaux O, Canlet C (2013). Relationships between the metabolome and the fatty acid composition of human saliva; effects of stimulation. Metabolomics.

[28] Schipper RG, Silletti E, Vingerhoeds MH (2007). Saliva as research material: Biochemical, physicochemical and practical aspects. Arch Oral Biol.

[29] Bongaerts J, Rossetti D, Stokes J (2007). The Lubricating Properties of Human Whole Saliva. Tribol Lett.

[30] Haward SJ, Odell JA, Berry M, Hall T (2011). Extensional rheology of human saliva. Rheol Acta.

[31] Francis CA, Hector MP, Proctor GB (2000). Precipitation of specific proteins by freeze-thawing of human saliva. Arch Oral Biol.

[32] Henderson TJ (2002). Quantitative NMR Spectroscopy Using Coaxial Inserts Containing a Reference Standard: Purity Determinations for Military Nerve Agents. Anal Chem.

[33] Singh MP, Saxena M, Saimbi CS, Arif JM, Roy R (2017). Metabolic profiling by ^1^H NMR spectroscopy of saliva shows clear distinction between control and diseased case of periodontitis. Metabolomics.

[34] Nicholson JK, Foxall PJ, Spraul M, Farrant RD, Lindon JC (1995). 750 MHz ^1^H and ^1^H-^13^C NMR spectroscopy of human blood plasma. Anal Chem.

[35] Al-Tarawneh SK, Border MB, Dibble CF, Bencharit S (2011). Defining salivary biomarkers using mass spectrometry-based proteomics: a systematic review. OMICS.

[36] Cheng Y-SL, Rees T, Wright J (2014). A review of research on salivary biomarkers for oral cancer detection. Clin Transl Med.

[37] Peterson BW, Sharma PK, van der Mei HC, Busscher HJ (2012). Bacterial cell surface damage due to centrifugal compaction. Appl Environ Microbiol.

[38] Rijsselaere T, Van Soom A, Maes D, de Kruif A (2002). Effect of centrifugation on in vitro survival of fresh diluted canine spermatozoa. Theriogenology.

[39] Schipper R, Loof A, de Groot J, Harthoorn L, Dransfield E, van Heerde W (2007). SELDI-TOF-MS of saliva: methodology and pretreatment effects. J Chromatogr B: Anal Technol Biomed Life Sci.

[40] Canty DJ, Zeisel SH (1994). Lecithin and Choline in Human Health and Disease. Nutr Rev.

[41] Emwas A-HM (2015). The strengths and weaknesses of NMR spectroscopy and mass spectrometry with particular focus on metabolomics research. Methods Mol Biol.

[42] Bell J, Brown J, Norman R, Sadler P, Newell D (1988). Factors affecting ^1^H NMR spectra of blood plasma: cancer, diet and freezing. NMR Biomed.

[43] Pinto J, Domingues MRM, Galhano E, Pita C, do CèuAlmeida M, Carreira IM, Gil AM (2014). Human plasma stability during handling and storage: impact on NMR metabolomics. Analyst.

[44] Yin P, Peter A, Franken H, Zhao X, Neukamm SS, Rosenbaum L, Lucio M, Zell A, Häring H-U, Xu G (2013). Preanalytical aspects and sample quality assessment in metabolomics studies of human blood. Clin Chem.

[45] Nicholson JK, Buckingham MJ, Sadler PJ (1983). High resolution ^1^H n.m.r. studies of vertebrate blood and plasma. Biochem J.

[46] Grootveld M, Silwood CJ (2005). ^1^H NMR analysis as a diagnostic probe for human saliva. Biochem Biophys Res Commun.

[47] Takahashi N, Washio J (2011). Metabolomic effects of xylitol and fluoride on plaque biofilm in vivo. J Dent Res.

[48] Lauridsen M, Hansen SH, Jaroszewski JW, Cornett C (2007). Human urine as test material in ^1^H NMR-based metabonomics: recommendations for sample preparation and storage. Anal Chem.

[49] Bernini P, Bertini I, Luchinat C, Nincheri P, Staderini S, Turano P (2011). Standard operating procedures for pre-analytical handling of blood and urine for metabolomic studies and biobanks. J Biomol NMR.

[50] Pramanik R, Thompson H, Kistler JO, Wade WG, Galloway J, Peakman T, Proctor GB (2012). Effects of the UK Biobank collection protocol on potential biomarkers in saliva. Int J Epidemol.

[51] Dawes C (1972). Circadian rhythms in human salivary flow rate and composition. J Physiol.

[52] Dallmann R, Viola AU, Tarokh L, Cajochen C, Brown SA (2012). The human circadian metabolome. Proc Natl Acad Sci U S A.

