# Supplementary material for: Developing and Standardizing a Protocol for Quantitative Proton Nuclear Magnetic Resonance (1H NMR) Spectroscopy of Saliva
Source: J Proteome Res. Author manuscript; Available in PMC 2019 Jun 11. (PMC6558279; doi:10.1021/acs.jproteome.7b00847)
Supplement: Supporting information [file EMS83073-supplement-Supporting_information.pdf]

Developing and Standardizing a Protocol for Quantitative Proton Nuclear Magnetic Resonance (^{1}H-NMR) Spectroscopy of Saliva

Alexander Gardner[†], Harold G. Parkes[‖], Guy H. Carpenter[†], Po-Wah So[⊥]

[†] Department of Mucosal and Salivary Biology, Dental Institute, King's College London, London, SE1 9RT, UK

[‖] Institute of Cancer Research, London, SW7 3RP, UK

[⊥] Department of Neuroimaging, Institute of Psychiatry, Psychology and Neuroscience, King's College London, Maurice Wohl Clinical Neuroscience Institute, 5, Cutcombe Road, London, SE5 9RX, UK

Corresponding author:

Email: po-wah.so@kcl.ac.uk   Tel: 020 7848 5453

Table of contents

Supplementary Figures:

Figure S-1: Factors affecting salivary metabolites - Exogenous substances.

Figure S-2: Factors affecting salivary metabolites - Intra-oral catabolism of dietary substances.

Figure S-3: Factors affecting salivary metabolites - Exercise induced changes.

Figure S-4: Comparison of 700 MHz CPMG and NOESY 1D ^{1}H-NMR spectra.

Figure S-5: Diagram of a NMR tube with a coaxial insert and the volume ratio calculation.

Figure S-6: Confirmation of assignment of acetoin in whole mouth unstimulated saliva.

Figure S-7: Investigation of the assignment of propylene glycol in whole mouth unstimulated saliva.

Figure S-8: Investigation of whole mouth saliva pre- and post-addition of propylene glycol.

Figure S-9: Comparison of different freeze-thaw treatments on the ^{1}H-NMR spectra of saliva.

Supplementary Tables:

Table S-1: Summary of selected salivary metabolite concentrations collected pre-, during and 2 h post-exercise.

Table S-2: Analysis of centrifugation force on metabolite concentrations.

Table S-3: Analysis of freeze-thaw considerations on metabolite concentrations.

Table S-4: Analysis of quantification method on metabolite concentrations.

Supplementary Information

Factors affecting salivary metabolites

Exogenous substances

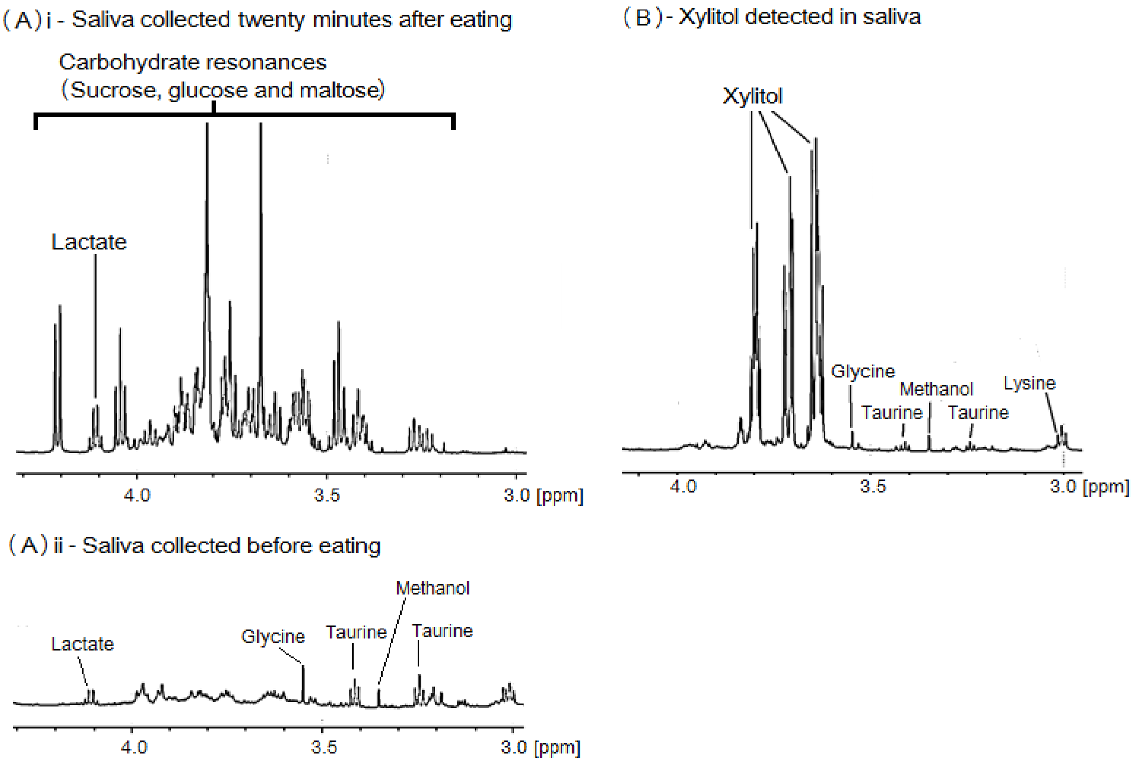

Figure S-1: (A) Partial 700 MHz CPMG ^{1}H-NMR spectra (echo time 64 ms, 3.00 – 4.10 ppm) of saliva collected from the same participant before and twenty minutes after eating. Spectra are of the same vertical scale. Peaks from sucrose, maltose and glucose obscure metabolites such as glycine and taurine and the quartet from lactate (4.12 ppm) in saliva collected twenty minutes after eating (i). These peaks were not observed in samples collected one hour after eating or drinking (ii). (B) Detection of xylitol from chewing gum in saliva collected one hour after chewing gum. Xylitol peaks did not obscure other assigned metabolites. Samples were centrifuged at 15,000 *g* prior to freezing.

Intra-oral catabolism of dietary substances

Certain exogenous substances not only obscure salivary NMR spectra but are readily metabolised in the oral cavity by the complex microbial community, and thus alterations in levels of other salivary metabolites can be observed. This is illustrated in Figure S2, showing the intra-oral catabolism of sucrose. Water (10 ml) was held in the mouth for 30 s before being expectorated. Saliva was then collected over a period of two minutes. This process was repeated after 5 mins with 0.25 M sucrose solution (10 ml).

Studies of saliva involving consumption of oral substances (including those administered as sialagogues, e.g. citric acid) therefore need to consider the effects these substances may have on metabolite profile.

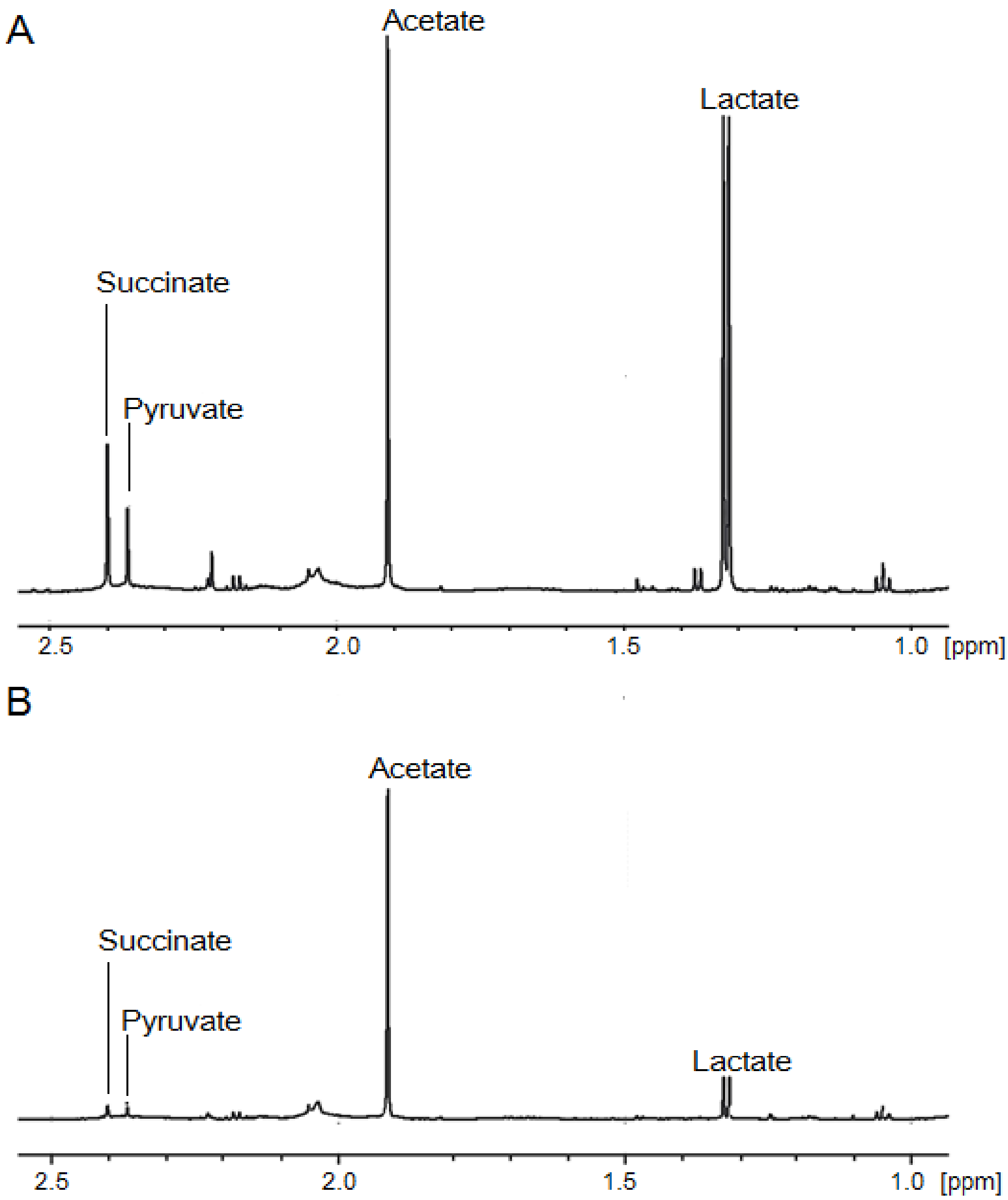

Figure S-2: Partial 700 MHz CPMG ^{1}H-NMR spectral regions (echo time 64 ms, 1.0 - 2.5 ppm) of saliva, expectorated following (A) a 0.25 M sucrose rinse and (B) a water rinse. Elevated acetate, lactate, pyruvate and succinate was observed in the saliva following a sucrose rinse. Samples were centrifuged at 15,000 *g* prior to freezing. Spectra are of the same vertical scale.

Exercise induced changes

Changes in salivary metabolite concentration were induced by exercise. Within ten minutes of continuous exercise, expectorated saliva had higher concentrations of all metabolites Levels returned to baseline within two hours post-exercise. The higher levels during exercise may partly be due to dehydration (i.e. less fluid leading to more concentrated metabolites), however metabolite concentrations do not change proportionally, indicating additional factors causing differential generation and consumption of metabolites. Recent exercise therefore presents an additional variable to consider prior to collecting saliva for ^{1}H-NMR spectroscopy.

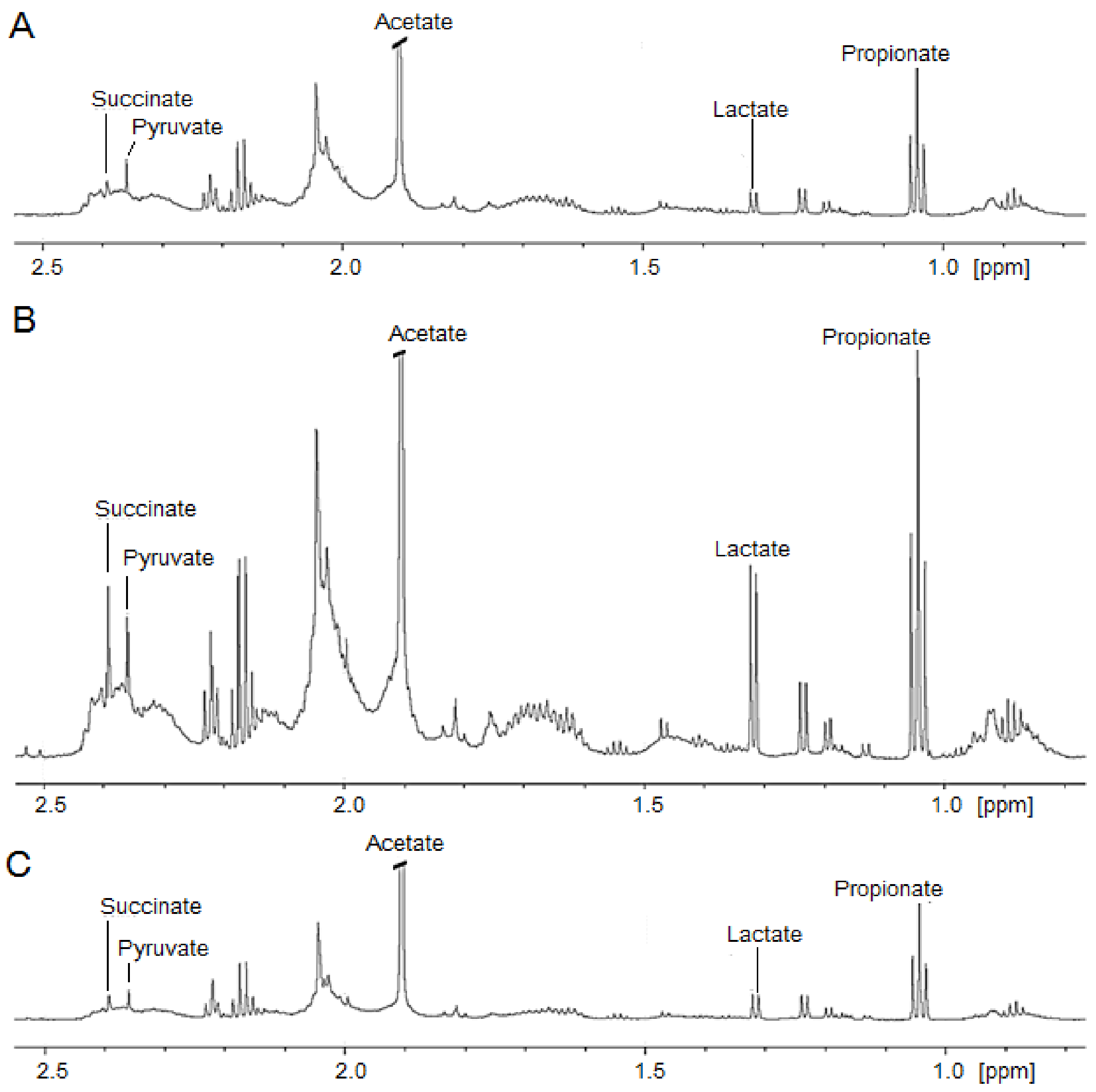

Figure S-3: Partial 700 MHz CPMG ^{1}H-NMR spectral regions (0.8 - 2.5 ppm) of saliva collected (A) before, (B) during and (C) two hours after exercise. Samples were centrifuged at 15,000 *g* prior to freezing, with quantification by internal, buffered TSP. Vertical scale is the same for all spectra. The acetate peak has been truncated.

Table S-1: Summary of selected salivary metabolite concentrations collected pre-, during and 2 h post-exercise (n=1), illustrating the disproportionate increases in salivary metabolites following exercise.

Metabolite	Pre-exercise (mM)	During exercise (mM)	Post-exercise (mM)
Acetate	2.08	6.46	2.26
Lactate	0.07	0.37	0.08
Propionate	0.29	0.72	0.29
Succinate	0.13	0.33	0.06
Pyruvate	0.08	0.43	0.09

Comparison of saliva with CPMG and NOESY pulse sequences

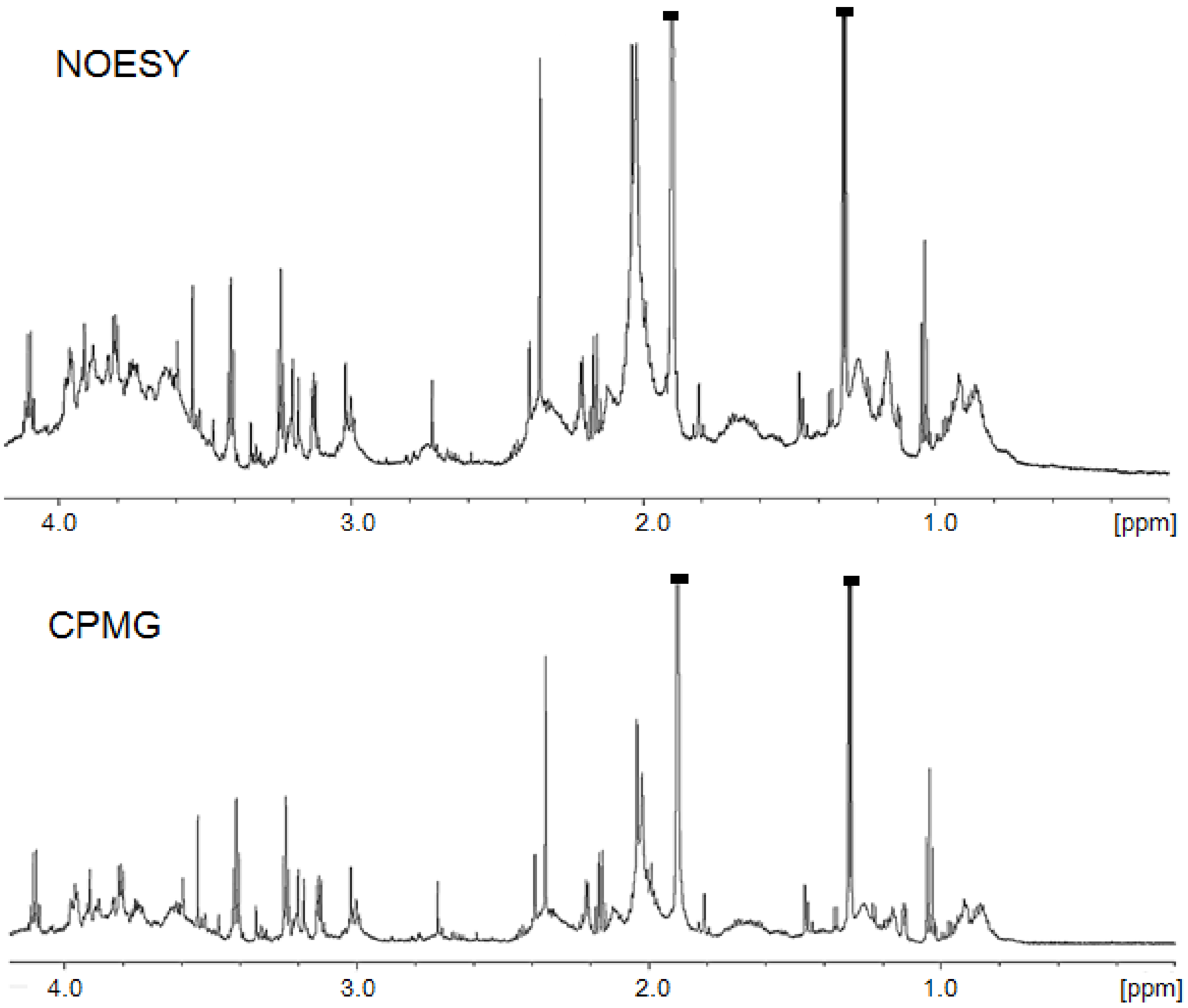

Figure S-4: Stacked partial spectra of partial 700 MHz ^{1}H-NMR spectral regions (0.1 – 4.1 ppm) of the same saliva sample analysed with a NOESY pulse sequence (top) and a CPMG pulse sequence (echo time 64 ms, bottom). Spectra are of the same vertical scale with the acetate and lactate peaks truncated. Spectra were similar, however, the CPMG spectra featured a flatter baseline than the NOESY spectrum, without attenuating the remaining resonances, and so the former was used for quantification. NOESY ^{1}H-NMR spectra were acquired at 700.13 MHz. 32 transients were collected with 64 k data points following four dummy scans, with a spectral width of 20 ppm (-5 to 15 ppm), a relaxation delay of 4 s and a mixing time of 10 ms.

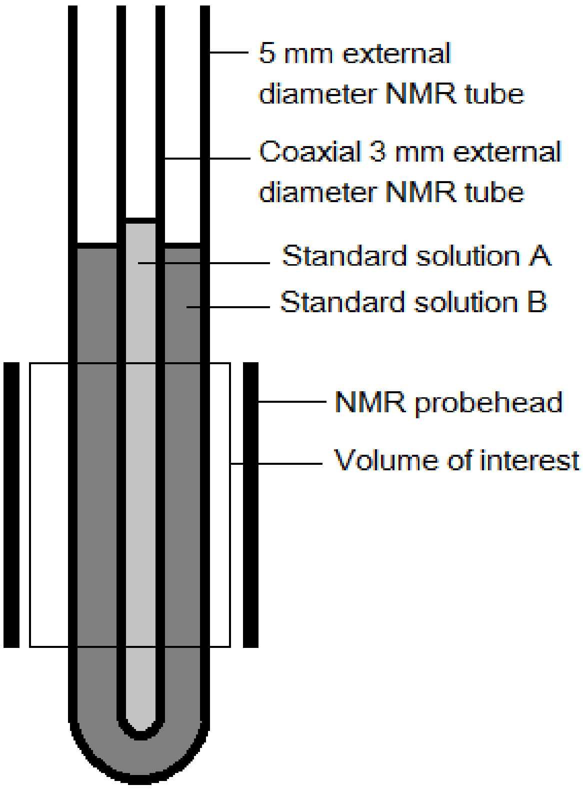

Figure S-5: Diagram to show how the volume ratio of a NMR tube and a coaxial insert was calculated. Different standard solutions (A and B) are placed into each tube. Both solutions are of known concentration, chemical shift, and number of protons giving rise to the peak to be integrated. Absolute volumes are not important, provided the volume read by the NMR probehead (rectangular area) is fully covered. Once the spectrum has been acquired and the peak integrals measured the volume ratio can be calculated using the equation:

$$\frac{Volume\ A}{Volume\ B} = \frac{Integral\ A}{Proton\ concentration\ A} * \frac{Proton\ concentration\ B}{Integral\ B}$$

Where proton concentration is the molar concentration of the solution multiplied by number of protons contributing to the peak that was integrated. Diagram is not to scale.

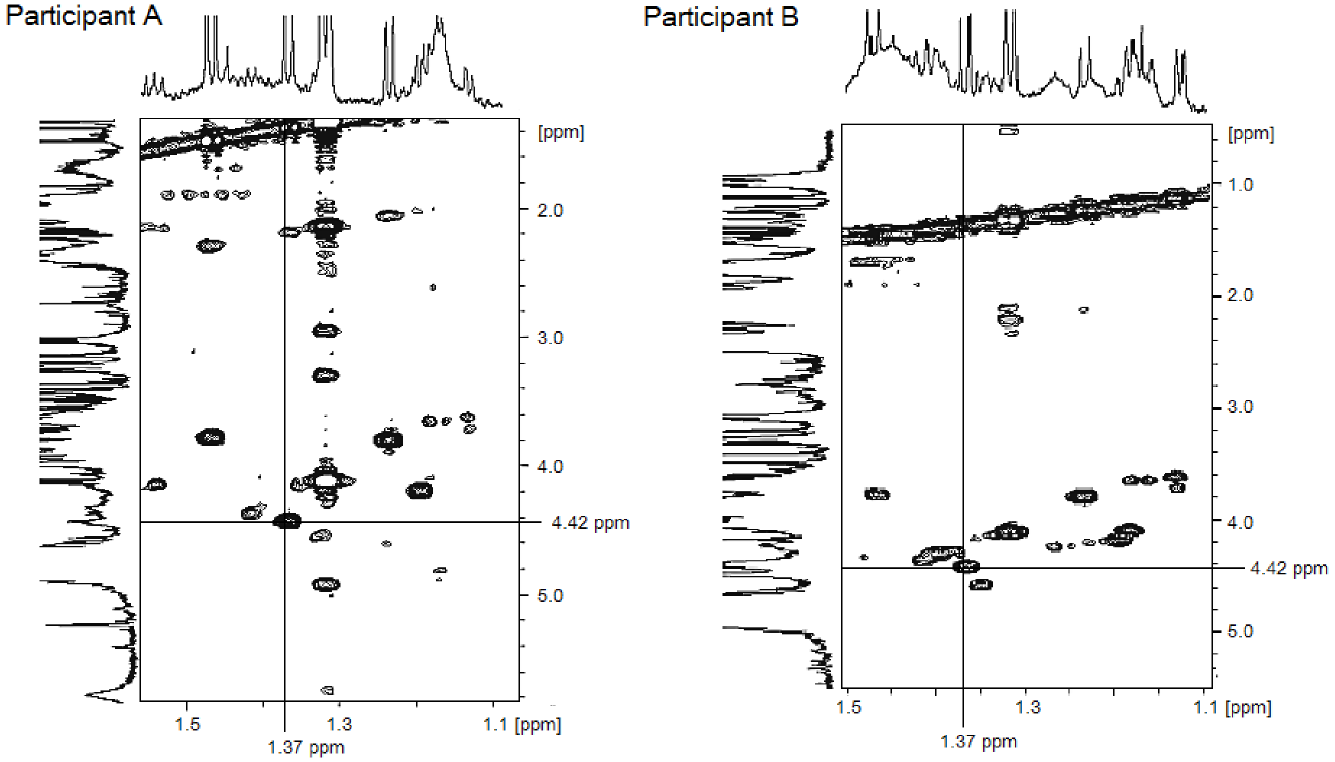

Figure S-6: Confirmed assignment of acetoin in whole mouth unstimulated saliva by 2D NMR. 2D ^{1}H-^{1}H COSY spectra were obtained from saliva samples of two participants, A and B. Spectra were acquired with 4096 data points, 400 increments, with 8 scans per increment, a relaxation delay of 2 s and spectral width 11,160 Hz (15.9 ppm). In both saliva samples, the doublet at 1.37 ppm (arising from the methyl group adjacent to the CH(OH) in acetoin) shows a cross peak at 4.42 ppm, which matches HMDB assignments for acetoin (http://www.hmdb.ca/spectra/nmr_one_d/1939). The quartet at 4.42 ppm from the proton in the CH(OH) group is masked by other resonances in the 1D ^{1}H-NMR spectra of saliva. Samples were centrifuged at 15,000 *g* prior to freezing, with quantification via external TSP in a coaxial tube.

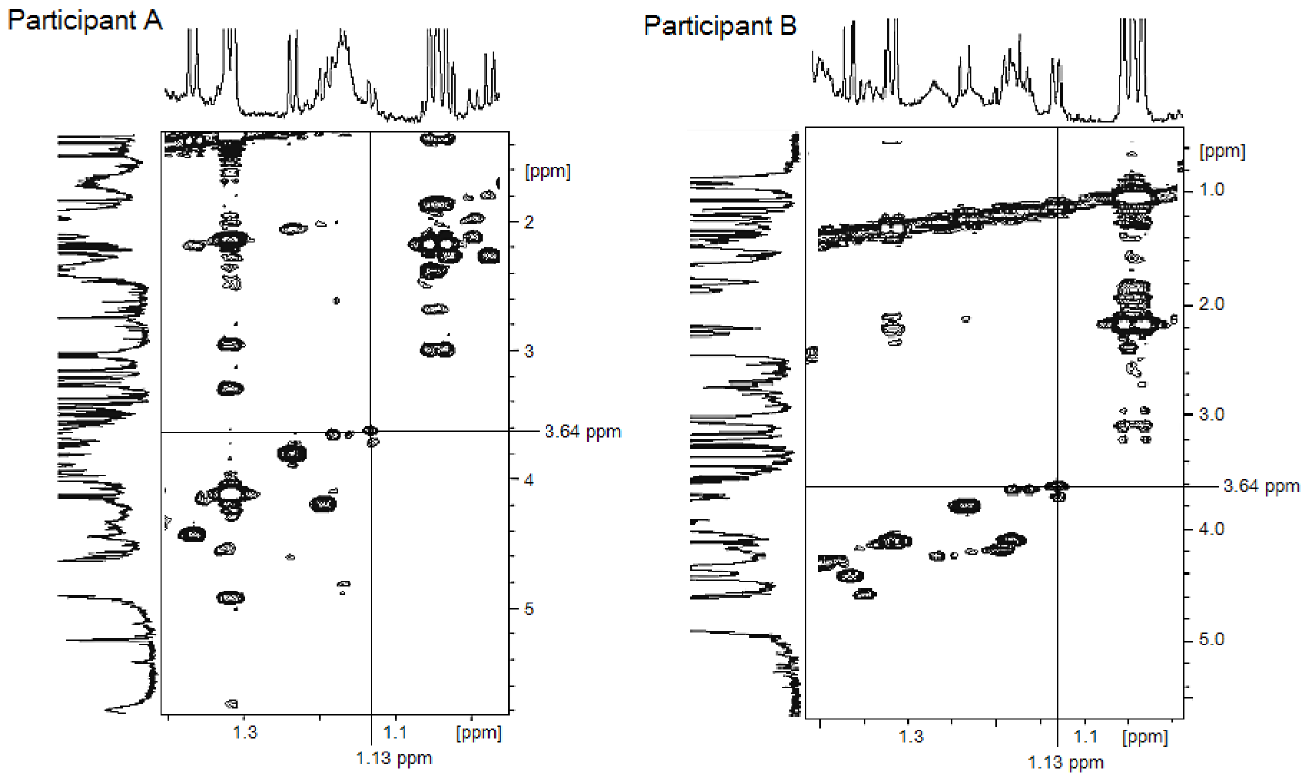

Figure S-7: Investigation of propylene glycol, reported in saliva by Singh *et al.*, 2017. 2D ^{1}H-^{1}H COSY spectra were obtained from the same saliva samples as described for Figure S-3. Cross peaks from the doublet at 1.13 ppm, believed to be propylene glycol, were observed at 3.64 ppm in both participants. This was not in line with the expected literature on propylene glycol (http://www.hmdb.ca/metabolites/HMDB01881) where the methyl group doublet at 1.13 ppm would display a cross peak with the adjacent CH group at 3.87 ppm. Additional investigation by spiking saliva with propylene glycol is described below (Figure S-5) to determine the assignment of propylene glycol in saliva by 2D NMR methods.

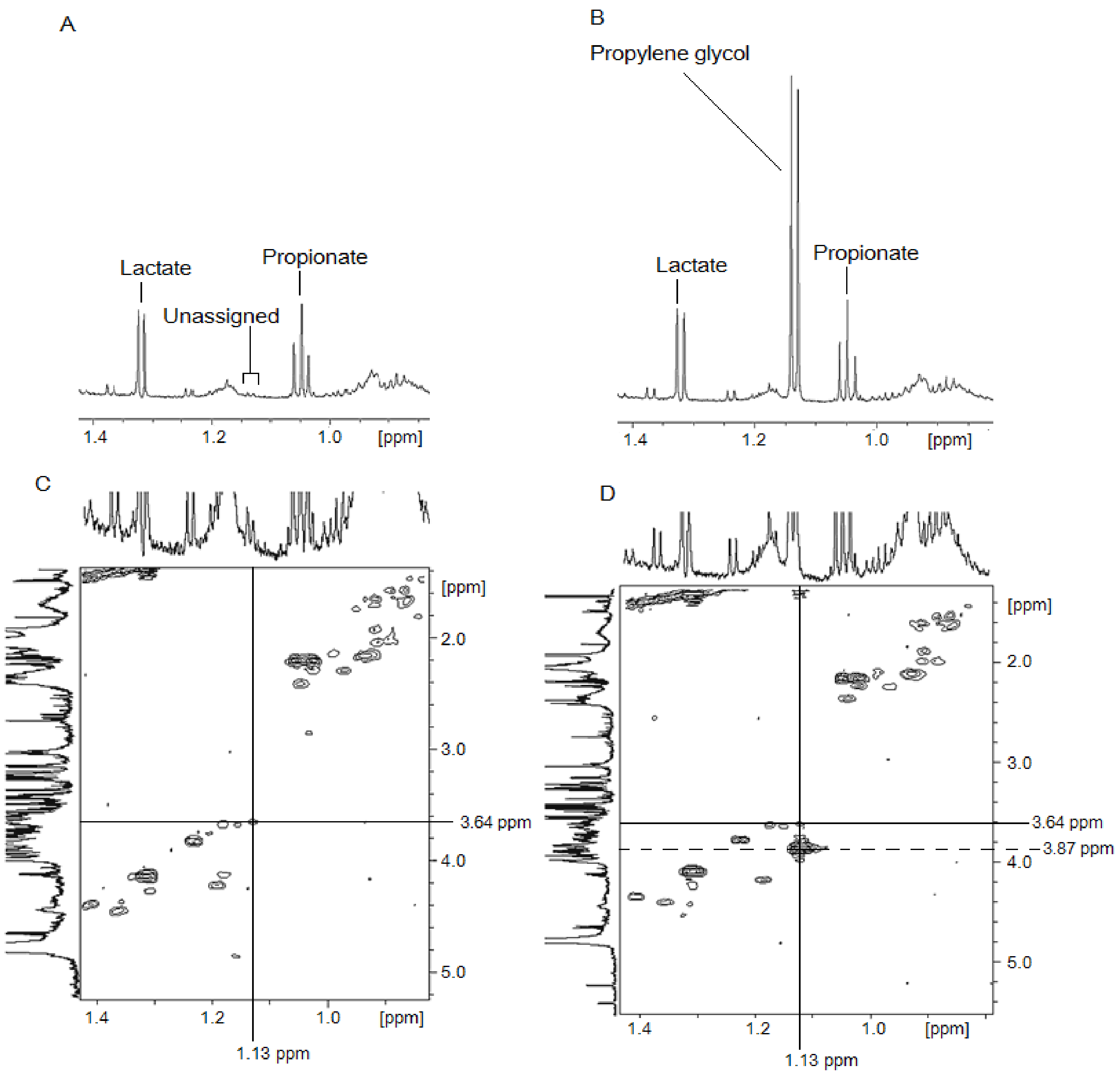

Figure S-8: Partial 700 MHz ^{1}H-NMR CPMG spectra of saliva displaying an unassigned doublet signal at 1.13 ppm, believed to be propylene glycol before (A) and after spiking in 0.1 mM propylene glycol, producing a doublet at the same frequency (B). The corresponding 2D ^{1}H-^{1}H COSY spectra shows a faint cross peak at 1.13 and 3.64 ppm, thought to be propylene glycol (C) but on addition of propylene glycol, another cross peak is evident at 1.13 and 3.87 ppm (D), suggesting the 1.13 ppm resonance in saliva does not arise from propylene glycol as previously assigned from the 1D-^{1}H NMR spectra.. Samples were centrifuged at 15,000 *g*, and analysed fresh. No standard was used as spectra were calibrated using the acetate peak. Spectra A and B are at the same vertical scale.

Comparison of different freeze-thaw treatments on the ^{1}H-NMR spectra of saliva

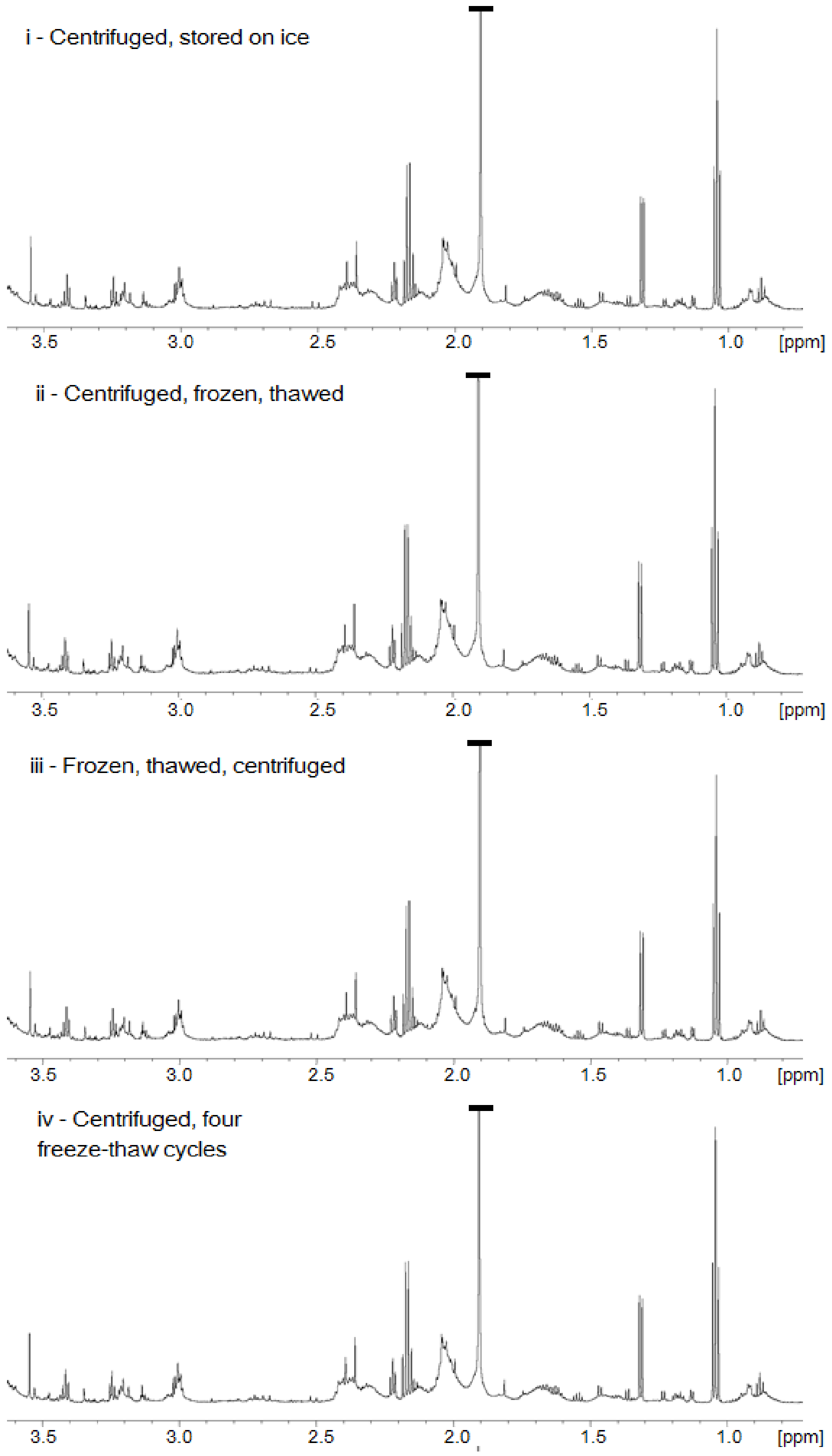

Figure S-9: Stacked partial spectra of partial 700 MHz CPMG ^{1}H-NMR spectral regions (0.6 - 3.6 ppm) of saliva sample collected at the same time from a representative individual. Spectra are of: (i) centrifuged at 15,000 *g* with no freezing; (ii) frozen and thawed after centrifuging at 15,000 *g*; (iii) frozen and thawed before centrifuging at 15,000 *g*; (iv) centrifuged at 15,000 *g* and then frozen and thawed four times. Quantification was via external TSP in a coaxial tube for all aliquots. The same degree of similarity was observed in the other participants. Spectra are of same vertical scale. The acetate peak has been truncated.

Table S-2: Analysis of centrifugation force on metabolite concentrations.

**Metabolite** (HMBD number)	**Metabolite concentration (mean ± SEM, µM) of sample centrifuged at:**					**Repeated measures ANOVA (p value) *= < 0.05**	**Bonferroni post-hoc test (groups compared; p value)**	**Bonferroni post-hoc test of normalised data (groups compared; p value)**
	**0 *g***	**750 *g***	**1500 *g***	**3000 *g***	**15,000 *g***			
Acetate (0000042)	3603.0 ± 318.8	3801.0 ± 388.5	3708.0 ± 348.0	3502.0 ± 250.0	3480.0 ± 279.9	0.26	N.S.	N.S.
Acetoin (0003243)	58.7 ± 9.2	43.9 ± 4.4	42.2 ± 4.0	39.3 ± 4.7	39.2 ± 4.8	0.03*	N.S.	N.S.
Alanine (0000161)	90.2 ± 8.8	84.3 ± 8.4	81.4 ± 8.1	79.7 ± 8.9	79.1 ± 9.1	0.039*	N.S.	N.S.
Butyrate (0000039)	215.2 ± 53.2	182.9 ± 34.9	169.2 ± 29.8	156.3 ± 22.0	152.4 ± 20.3	0.14	N.S	N.S.
Choline and choline-containing compounds (0000097)	11.1 ± 2.2	10.2 ± 1.9	10.0 ± 2.0	9.5 ± 2.1	9.8 ± 2.0	0.013*	N.S.	0*g* vs. 1500*g*; 0.019
Citrate (0000094)	35.1 ± 4.9	35.4 ± 4.3	36.5 ± 4.6	32.0 ± 4.4	35.0 ± 4.9	0.34	N.S.	N.S.
Dimethylamine (0000087)	6.8 ± 0.8	7.1 ± 1.1	6.8 ± 0.9	6.3 ± 0.9	6.6 ± 1.1	0.16	N.S.	N.S.
Ethanol (0000108)	74.2 ± 13.1	75.6 ± 13.1	70.7 ± 11.7	69.3 ± 12.6	67.5 ± 11.6	0.079	N.S.	N.S.
Formate (0000142)	88.6 ± 65.0	111.0 ± 73.7	104.4 ± 70.6	90.0 ± 57.9	91.1 ± 59.5	0.25	N.S.	N.S.
Glycine (0000123)	131.7 ± 19.8	132.9 ± 21.3	133.2 ± 20.8	127.5 ± 20.6	130.2 ± 21.5	0.26	N.S.	N.S.
Histidine (0000177)	36.3 ± 6.1	36.9 ± 5.8	36.9 ± 5.6	35.0 ± 6.4	37.1 ± 6.2	0.32	N.S.	N.S.
Lactate (0000190)	238.4 ± 57.8	171.9 ± 37.3	159.1 ± 32.0	151.3 ± 37.2	144.1 ± 34.5	0.015*	N.S.	0*g* vs. 750*g*; 0.024<br>0*g* vs. 3000*g*; 0.012<br>0*g* vs. 15,000*g*; 0.008
Methanol (0001875)	30.6 ± 4.3	33.3 ± 4.3	32.8 ± 4.3	30.6 ± 3.4	31.3 ± 3.9	0.22	N.S.	N.S.
Methylamine (0000164)	9.2 ± 1.0	9.6 ± 0.9	9.4 ± 1.0	8.6 ± 1.3	8.8 ± 1.2	0.20	N.S.	N.S.
Phenylalanine (0000159)	41.5 ± 5.0	41.4 ± 4.9	41.9 ± 5.6	39.3 ± 5.1	34.6 ± 6.5	0.29	N.S.	N.S.
Propionate (0000237)	523.0 ± 130.9	564.2 ± 134.1	549.6 ± 127.3	498.4 ± 89.4	492.2 ± 92.3	0.25	N.S.	N.S.
Pyruvate (0000243)	122.8 ± 19.7	119.8 ± 18.6	119.9 ± 19.5	116.9 ± 19.5	119.7 ± 20.9	0.40	N.S.	N.S.
Succinate (0000254)	69.7 ± 13.7	72.3 ± 14.8	71.1 ± 14.2	67.5 ± 13.4	69.4 ± 13.8	0.24	N.S.	N.S.
Taurine (0000251)	158.5 ± 28.9	161.0 ± 32.7	156.3 ± 28.2	150.6 ± 31.4	153.6 ± 32.5	0.46	N.S.	N.S.
Tyrosine (0000158)	35.7 ± 3.5	34.6 ± 3.1	34.0 ± 2.9	32.7 ± 3.2	28.6 ± 4.4	0.29	N.S.	N.S.
Trimethylamine (0000906)	2.7 ± 0.5	2.8 ± 0.5	2.7 ± 0.6	2.6 ± 0.6	2.6 ± 0.6	0.46	N.S.	N.S.

Table S-3: Analysis of freeze-thaw considerations on metabolite concentrations.

**Metabolite** (HMBD number)	**Metabolite concentration (mean ± SEM, µM) of sample subject to freeze-thaw treatment:**				**Repeated measures ANOVA (p value) *= < 0.05**	**Bonferroni post-hoc test (groups compared; p value)**	**Bonferroni post-hoc test of normalised data (groups compared; p value)**
	Centrifuged, not frozen	Centrifuged, frozen, thawed	Frozen, thawed, centrifuged	Centrifuged, four freeze-thaw cycles			
Acetate (0000042)	3366.0 ± 398.1	3478.0 ± 453.0	3358.0 ± 406.0	3560.0 ± 445.4	0.14	N.S.	N.S.
Acetoin (0003243)	40.5 ± 5.3	40.4 ± 5.2	38.6 ± 4.8	40.2 ± 5.3	0.04*	N.S.	N.S.
Alanine (0000161)	107.4 ± 24.9	106.0 ± 23.6	107.7 ± 23.6	106.5 ± 23.1	0.58	N.S.	N.S.
Butyrate (0000039)	171.0 ± 23.4	169.2 ± 22.3	165.1 ± 20.5	167.1 ± 20.8	0.44	N.S.	N.S.
Choline and choline-containing compounds (0000097)	14.3 ± 3.5	14.1 ± 3.4	14.4 ± 3.5	14.2 ± 3.5	0.66	N.S.	N.S.
Citrate (0000094)	57.4 ± 13.1	54.4 ± 12.2	57.5 ± 13.2	55.7 ± 11.4	0.44	N.S.	N.S.
Dimethylamine (0000087)	13.2 ± 3.1	13.2 ± 3.2	12.8 ± 3.0	12.9 ± 2.8	0.50	N.S.	N.S.
Ethanol (0000108)	79.2 ± 13.3	80.2 ± 13.5	76.6 ± 11.9	80.6 ± 12.9	0.23	N.S.	N.S.
Formate (0000142)	80.0 ± 28.1	81.3 ± 28.7	68.7 ± 25.8	84.9 ± 31.9	0.14	N.S.	N.S.
Glycine (0000123)	143.2 ± 24.8	142.7 ± 24.7	142.2 ± 24.1	144.4 ± 23.5	0.42	N.S.	N.S.
Histidine (0000177)	19.7 ± 5.2	18.0 ± 4.6	19.6 ± 5.1	18.5 ± 5.0	0.14	N.S.	N.S.
Lactate (0000190)	333.1 ± 85.2	334.8 ± 86.2	326.0 ± 84.2	344.9 ± 87.6	0.17	N.S.	N.S.
Methanol (0001875)	20.6 ± 3.3	20.2 ± 3.2	20.6 ± 3.3	21.9 ± 3.8	0.18	N.S.	N.S.
Methylamine (0000164)	8.9 ± 1.7	9.2 ± 1.6	8.7 ± 1.5	8.8 ± 1.4	0.32	N.S.	N.S.
Phenylalanine (0000159)	53.9 ± 10.5	51.5 ± 9.9	53.4 ± 9.6	52.5 ± 9.6	0.36	N.S.	N.S.
Propionate (0000237)	468.9 ± 109.8	489.5 ± 114.9	468.6 ± 108.5	498.9 ± 114.1	0.19	N.S.	N.S.
Pyruvate (0000243)	115.0 ± 22.8	115.2 ± 23.0	116.4 ± 23.5	115.6 ± 22.6	0.81	N.S.	N.S.
Succinate (0000254)	222.5 ± 48.0	217.9 ± 46.4	215.3 ± 43.4	218.8 ± 46.4	0.38	N.S.	N.S.
Taurine (0000251)	209.2 ± 39.7	213.4 ± 39.0	217.2 ± 39.9	220.6 ± 42.5	0.25	N.S.	N.S.
Tyrosine (0000158)	40.0 ± 10.4	39.9 ± 10.4	41.8 ± 11.0	41.2 ± 10.6	0.07	N.S.	N.S.
Trimethylamine (0000906)	2.7 ± 0.6	2.7 ± 0.5	2.7 ± 0.6	2.6 ± 0.5	0.28	N.S.	N.S.

Table S-4: Analysis of quantification method on metabolite concentrations.

**Metabolite** (HMBD number)	**Metabolite concentration (mean ± SEM, µM) of sample quantified by:**			**Repeated measures ANOVA (p value) *= < 0.05**	**Bonferroni post-hoc test (groups compared; p value)**
	Internal, buffered TSP	Internal, unbuffered TSP	External TSP		
Acetate (0000042)	3336.0 ± 380.8	2990.0 ± 194.7	3480.0 ± 279.9	0.05*	External vs. internal, unbuffered; 0.049
Acetoin (0003243)	38.9 ± 4.2	38.6 ± 4.6	38.8 ± 4.5	0.97	N.S.
Alanine (0000161)	73.8 ± 9.2	72.5 ± 7.3	77.9 ± 9.2	0.31	N.S.
Butyrate (0000039)	154.2 ± 25.9	142.2 ± 18.1	149.3 ± 20.9	0.38	N.S.
Choline and choline-containing compounds (0000097)	9.2 ± 1.8	9.5 ± 1.8	9.8 ± 2.0	0.61	N.S.
Citrate (0000094)	31.9 ± 5.4	30.6 ± 3.9	33.7 ± 4.6	0.34	N.S.
Dimethylamine (0000087)	6.1 ± 1.0	5.9 ± 0.8	6.5 ± 1.1	0.28	N.S.
Ethanol (0000108)	101.1 ± 31.9	90.4 ± 22.5	99.3 ± 28.1	0.35	N.S.
Formate (0000142)	87.6 ± 61.8	68.8 ± 45.4	88.7 ± 59.0	0.26	N.S.
Glycine (0000123)	125.5 ± 21.3	122.0 ± 16.6	128.9 ± 21.4	0.59	N.S.
Histidine (0000177)	35.8 ± 6.3	36.0 ± 5.8	38.5 ± 6.6	0.57	N.S.
Lactate (0000190)	124.7 ± 24.7	129.8 ± 32.8	144.1 ± 34.5	0.37	N.S.
Methanol (0001875)	31.9 ± 4.5	27.7 ± 3.4	31.0 ± 4.0	0.012*	External vs. internal, unbuffered; 0.024
Methylamine (0000164)	9.1 ± 1.3	7.5 ± 0.9	8.8 ± 1.2	0.07	N.S.
Phenylalanine (0000159)	40.7 ± 5.2	42.5 ± 5.0	39.6 ± 4.9	0.32	N.S.
Propionate (0000237)	491.0 ± 120.7	456.5 ± 120.7	492.2 ± 92.3	0.53	N.S.
Pyruvate (0000243)	113.0 ± 19.4	112.4 ± 20.8	119.3 ± 21.0	0.53	N.S.
Succinate (0000254)	146.2 ± 28.8	148.4 ± 20.8	143.1 ± 26.9	0.77	N.S.
Taurine (0000251)	139.4 ± 25.8	138.8 ± 36.8	151.9 ± 33.1	0.50	N.S.
Tyrosine (0000158)	29.3 ± 3.3	29.7 ± 2.3	30.3 ± 2.9	0.73	N.S.

Trimethylamine (0000906)	2.4 ± 0.6	2.6 ± 0.6	2.4 ± 0.6	0.22	N.S.
